# The ‘light touch’ of the Black Death in the Southern Netherlands: an urban trick?†

**DOI:** 10.1111/ehr.12667

**Published:** 2018-02-05

**Authors:** Joris Roosen, Daniel R. Curtis

**Affiliations:** ^1^ Utrecht University; ^2^ Leiden University

## Abstract

Although the fanciful notion that the Black Death bypassed the Low Countries has long been rejected, nevertheless a persistent view remains that the Low Countries experienced only a ‘light touch’ of the plague when placed in a broader European perspective, and recovered quickly and fully. However, in this article an array of dispersed sources for the Southern Netherlands together with a new mortmain accounts database for Hainaut show that the Black Death was severe, perhaps no less severe than other parts of western Europe; that serious plagues continued throughout the fourteenth and fifteenth centuries; and that the Black Death and recurring plagues spread over vast territories—including the countryside. The previous conception of a ‘light touch’ of plague in the Low Countries was created by the overprivileging of particular urban sources, and a failure to account for the rapid replenishment of cities via inward migration, which obscured demographic decimation. We suggest that the population of the Low Countries may not have recovered faster than other parts of western Europe but instead experienced a greater degree of post‐plague rural–urban migration.

In the 1950s, a fanciful notion was perpetuated that much of the Low Countries, especially the south, was remarkably unscathed by the Black Death of 1347–52.^1^ Subsequent works have shown this to be a complete fallacy.^2^ Nevertheless, there is still a persistent perception that the Low Countries escaped the full brunt of plague. Skilled work with manorial sources for England and fiscal sources for Italy and France has estimated 30–70 per cent population losses in some localities after the Black Death,^3^ with high mortality rates also caused by fifteenth‐century epidemics.^4^ Yet while broad aggregated ‘country‐level’ data (which must be viewed cautiously) have suggested that places such as Ireland and Scandinavia lost roughly 50 per cent of their population, in the Southern Netherlands losses were apparently less than 15 per cent.^5^ In the 1960s, a famous map by Carpentier identified the Low Countries as one of the few places in Europe where plague was basically absent,^6^ and remarkably, this map has been reproduced uncritically, even in recent works.^7^ International literature makes frequent reference to the ‘light touch’ of the Black Death in the Low Countries: we learn from Cohn and the co‐authored work of Scott and Duncan, for example, that Douai and Liège largely escaped the initial outbreak.^8^ Accordingly, Blockmans et al. suggest that ‘losses [in the Low Countries] were not as high as the 25 to 40 per cent assumed for England and Italy’,^9^ while Christakos et al. claim that ‘unlike Northern Italy, mortality in Belgium was the lowest among present‐day countries, with about 20 per cent mortality at most’.^10^

As even specialists of the medieval Low Countries downplay plague severity, especially when viewed comparatively across Europe, it is unsurprising that similar ideas have filtered through to the general literature. Some take the view that early fourteenth‐century famines were more damaging than the Black Death,^11^ and those conceding higher plague severity tend to argue that the worst of it occurred in the echo‐epidemics of the later fourteenth and even fifteenth centuries.^12^ The demographic devastation depicted by Abel for Germany in the wake of the Black Death, including totally deserted villages known as *Wüstungen*, was thought to be basically absent from inland Flanders, Hainaut, and Brabant,^13^ though some lost settlements and farms did appear in coastal Flanders.^14^ In his authoritative synthesis on medieval economy and society in the Low Countries, van Bavel clearly states that ‘Flanders was not hit hard at all’, and that population levels in 1400 were close to those in 1300.^15^ Likewise, Holland ‘did not suffer great loss of life’.^16^

This view of a ‘light touch’ of the Black Death in the Low Countries has been made all the more convincing by the notion that population recovery was apparently so rapid and full. While it is claimed that the population in parts of the Low Countries recovered to its pre‐plague levels during the fifteenth century,^17^ this did not take place until the sixteenth century in the Italian and Iberian peninsulas and not until the eighteenth century in parts of England.^18^ As early as the 1970s, Pounds pointed to an association between low mortality and quick recovery, noting that ‘the Low Countries appear to have been visited less severely by the plague than many other areas, and it is to be presumed that the population recovered more quickly’,^19^ a view taken up in recent works, where it was noted that ‘in the Low Countries … population recovered much faster in part because the impact of the Black Death was more limited’.^20^ In the north, the ‘jump‐start’ of Holland's economy is said to have been built upon a comparatively mild outbreak of plague interacting with an already capital‐intensive path of production, a frontier economy with low Malthusian pressures on resources, and freedoms for producers to respond to changing exogenous pressures, leading to ‘strong [population] growth during the fifteenth century [which] induced … convergence of (nominal and real) wage levels’.^21^

The ‘light touch’ story is problematic, however, because we have no convincing explanation for why the Black Death seemingly exhibited such weak effects in the Low Countries. In fact, it represents an ‘epidemiological conundrum’, especially in the heart of the Southern Netherlands, which was unusually densely populated, heavily urbanized, and intensely commercialized, as well as being a battleground during the Hundred Years’ War. Tentative explanations have been offered, such as the early dominance of nuclear household structures, with each household becoming its own ‘cordon sanitaire’,^22^ yet such explanations carry little weight given that nuclear households also characterized other parts of north‐western Europe that were hit hard by the Black Death. Dhérent argues that the city of Douai, for example, avoided the worst effects of the Black Death by introducing superior quarantining measures and hygiene regulations.^23^ Once again we must question the logic here: later outbreaks of plague were apparently severe in Douai, despite little change in the nature and implementation of these regulations. Slicher van Bath links the quality of nutrition seen in the Low Countries with greater capacity to withstand the plague.^24^ However, much literature now suggests that malnutrition and plague are not linked causally,^25^ and second, if the theory were correct, we would expect the Southern Netherlands to have weaker resistance given that this region experienced some of the worst conditions in the Great Famine of 1315–17 and subsequent subsistence crises.^26^

In light of these discrepancies, this article empirically reviews the notion that the Low Countries were only lightly affected by the Black Death. It focuses explicitly on the difficult case of the Southern Netherlands. In section I, we concentrate on some problems and limitations with the evidence presented in earlier studies, and how this may have fostered a conception of the Southern Netherlands as escaping the worst effects of the Black Death. In section II, we use dispersed archival data and a new database of mortmain accounts to argue that the Black Death took a severe toll on the Low Countries’ population; an upward revision of mortality in line with recent research elsewhere in Europe.^27^ We also argue that the severe plagues that recurred throughout the fourteenth and fifteenth centuries, rather than being evidence for the weakness of the initial Black Death, simply exacerbated a harsh late medieval mortality regime. In section III, we show that the Black Death and later epidemics afflicted the countryside too, sometimes as severely as the cities. In section IV, we show that the traditional view of a ‘light touch’ of plague was the result of historians overprivileging urban sources that do not account for the rapid replenishment of cities via inward migration—and thus hiding true demographic devastation: the Southern Netherlands in particular was adept at recovering damaged urban agglomerations. In the conclusion we suggest that the relatively rapid and full late medieval population recovery in the Low Countries still needs to be demonstrated empirically, and that, if it did indeed occur, it was not connected to reduced mortality but potentially increased rates of nuptiality and fertility.

## I

The lack of source material to quantify the demographic impact of plague in the fourteenth and fifteenth centuries is a problem across Europe.^28^ Sources for England and Italy, for example, are more abundant, but still nothing exists that allows for a reconstruction of the epidemiological characteristics of plague over large areas. Scholars tend to extrapolate from localized micro‐studies (some manors in England, for example), or calculate mortality rates from indirect data.^29^ For the late medieval Low Countries, demographic sources are not plentiful.^30^ However, the situation is better for the south than the north: fiscal inquiries and hearth counts found for fourteenth‐ and fifteenth‐century Flanders, Hainaut, Brabant, and Artois do not survive in similar quantities for the Northern Netherlands, with the Duchy of Guelders a notable exception.^31^ That is not to say these sources are perfectly suitable for assessing plague mortality: many hearth counts have large chronological gaps between assessments, few go back before the Black Death, and we have no reliable figure for the numbers of households excluded from registration, on account of poverty for example.

Another problem is the difficulty in discerning whether a decline in hearths should be attributed to mortality or to other demographic variables, such as declining nuptiality and fertility, or migration. A specific problem related to calculating plague mortality is the fact that the hearth assessment was based on whole households. Plague mortality does not always show up clearly in hearth counts because plague epidemics did not always kill every household member. Recent evidence shows that household contagion was a feature of plague (with family members dying in quick succession),^32^ though this did not mean that the Black Death always finished off whole households. Therefore, any work that bases plague mortality on these sources probably understates its severity.

These challenges do not only apply to demographic sources: according to Noordegraaf and Valk, direct references to plague in narrative or administrative sources are scarce in the period before 1550, though their work was limited to Holland.^33^ One thing that is certain is that sources are less forthcoming during the Black Death in the Low Countries than for other periods in the fourteenth century. This problem manifests itself in two ways, further entrenching the ‘light touch’ narrative. First, where sources exist, they often obscure or downplay societal, economic, or political disruption. Derville notes that the accounts of the city of Lille in 1349 seemed on the surface to indicate life continuing as normal, but after more scrupulous inspection it became clear that during the epidemic the city gates were opened each night to allow municipal chariots to carry the dead to mass graves in the suburbs.^34^ As suggested recently by Rommes, urban governments tried to give an impression that everything was functioning as normal and attempted to maintain trading networks with other cities and the hinterlands—to do otherwise was to risk economic ruin.^35^

Second, there is the likelihood that the Black Death actually contributed to the lack of sources. Missing sources are not necessarily an indicator of the absence of the epidemic, but could be caused by the administrative breakdown it instigated.^36^ The notion that the Black Death bypassed the town of Douai, for example, has been predicated on the lack of wills produced at that time, in complete contrast to the massive spike in the epidemic of 1400–1, supporting the view that echo‐epidemics were more substantial in the Low Countries.^37^ However, in reality we are not talking about a low number of wills during the Black Death but almost none at all, which suggests a breakdown in administrative procedure instead. Similarly there were no wills recorded for the early and late 1360s, despite plagues being attested for these periods across the whole of the Low Countries. Another reason may be that during the Black Death outbreak, people were not yet used to the precise symptoms of the disease, and so did not necessarily realize the precariousness of their situation—hence the lack of will writing. Only in later plague outbreaks did people become acquainted with these indicators of impending death.^38^ Comparable trends can also be seen in the numbers of probate inventories recorded from 1349 to 1440 in Ghent, for example, where significant spikes are seen for the repeat plague outbreaks but not for the Black Death.^39^ Again we have to question the administrative process here: in 1349, the year when hyper‐mortality takes off (as demonstrated below), the number of probate inventories is the lowest of all years up to 1440, reaching no more than one‐fifth of the value of even the ‘normal’ years.

## II

The paucity and limitations of the sources may have led to the ‘light touch’ view of plague in the Low Countries. However, recent archival research has begun to uncover evidence contrary to this established view. In this section we bring together these dispersed pieces into a more coherent whole, alongside our own archival evidence. First we look at the Black Death, before turning our attention to the repeat outbreaks across the fourteenth and fifteenth centuries.

One of the places to which much recent research has been dedicated is the city of Bruges. In 1952, Rogghé offered as evidence for plague a letter of permission from Count Louis II of Flanders to construct two new graveyards outside the city walls on 15 August 1349,^40^ and this evidence has been supported recently by the discovery of an additional document which shows that by 18 July 1349, the existing graveyard at St Jacob's Church had already been enlarged.^41^ However, as van Werveke pointed out, this is not evidence for plague mortality per se: it could also be evidence for precautionary measures dictated by fear of a potential outbreak. Indeed, in England, the London cemetery of East Smithfield was established in anticipation of plague mortality, not in response to actual pressures.^42^ In place of this anecdotal information, however, quantifiable evidence has been provided. In 1980, Maréchal exploited the archive of the ‘*Potterie*’ hospital of St John in Bruges to show that the entire nursing staff perished during the Black Death, while there was a seven‐fold increase in the admissions of new members.^43^ Given that new admissions took place only when there were vacancies, this is a good proxy for mortality: using this information Vandeburie calculated a mortality rate of 35 per cent at the institution.^44^ Maréchal also employed a larger dataset of annuities, from which she calculated a mortality rate of 11 per cent among annuitants in the orphanage books of Bruges by cross‐referencing names in the city accounts.^45^ This estimate, however, has been shown to be rather low. More recently, Vandeburie compared the number of expired annuities (13) with the total number of annuitants (42) at St John's hospital to calculate a mortality rate of 31 per cent between May 1349 and May 1350.^46^

Information for other cities is not as plentiful as for Bruges, but taken together the fragments are suggestive of higher mortality than previously supposed. Using the registers of tutelage and orphanage accounts, Blockmans noted that mortality was higher than normal between 1349 and 1352 in Ghent.^47^ In the accounts of the St Nicholas Church, employing an overview of parishioners entitled to a dividend from the Table of the Holy Spirit, Vermeersch recently found that the account for October 1348 to October 1349 had 36 names with seven crossed out (indicating death), while in the account of 1346–7 only one of 30 names was crossed out. Similarly in a list of 39 annuitants of the deanery of St Bavo running from June 1349 to June 1350, six were given the sign of a cross.^48^ Elsewhere, Ypres was one of the few cities in the older literature actually said to have been hit relatively hard by the plague, and recent evidence of a tripling of the number of issue payments between October 1349 and October 1350 in the city accounts corroborates this.^49^ As already mentioned, Douai was a city thought to have escaped the Black Death, though a re‐examination of the sources by Derville indicated that in these years a third of the city's aldermen died, as did six out of nine vicars of the college of Saint‐Amé.^50^ Derville also uncovered further information for Lille showing that in the hospital of St Saveur, more than two‐thirds of its city debtors and more than a third of its debtors in the rural hinterlands disappeared from records in the period 1349–54.^51^ The blaming and burning of Jews in accounts dating from 5 August 1349 also does not suggest a modest impact.^52^ In St‐Omer in 1349, 33 money‐changers were said to exist within the city, but in 1352 only 13 remained: a loss of 61 per cent.^53^ Just 10 kilometres further east, in the small town of Aire‐sur‐la‐Lys, the amount of escheated property of bastards multiplied by nine in 1350.^54^

Although fragmentary, this provides a new picture of plague severity in the initial outbreak: frequently the sources point to at least a 30 per cent loss. This was not the end, however, as the effects of this shock were further enhanced by recurrent severe plague outbreaks across the late medieval Southern Netherlands, contributing to a rather harsh late medieval mortality regime. The abbot of St‐Bertin abbey in St‐Omer reported in September 1361, for example, that the ‘plague attacked day after day causing a great pestilence and mortality’. Between 1357 and 1366, the abbey experienced a decline in communicants of nearly half, from 10,200 to 5,350.^55^ Churches in other parishes had similar drops: from 2,500 to 1,200 (St‐Denis), from 2,600 to 1,300–1,400 (St‐Sépulcre), from 1,800 to 1,000 (St‐Aldegonde), and from 3,300 to 1,800 (St‐Margueritte).^56^ Cities such as Lille had severe afflictions during the outbreak of the late 1360s: the steepest spike in mortality among rentiers came during the period 1368–9.^57^ Similarly in Ghent, the number of coffins purchased by the Table of the Holy Spirit of St Nicholas Church increased substantially during both plague periods of 1361–2 and 1367–8 to six and 11, respectively, from effectively one per year (and often none at all) in normal times.^58^

Many cities showed signs of exceptionally severe mortality during the plague of 1400–1: a general phenomenon seen everywhere in the Low Countries. The hospital of St‐Jean at Arras buried 246 bodies from 1 October 1399 to 30 September 1400, while from 1371–3 they had buried no more than 50 per year.^59^ The plague in 1414–15 also raised burials to around 250.^60^ In Mons, the accounts from St Jan Baptist's ‘great alms’ show 438 coffins purchased in 1400–1, compared to an annual non‐plague average of roughly 50.^61^ The number of new wills reached a peak in 1400 at Douai. In nearby Tournai, 339 wills were found for the year 1400, while a normal year never saw more than 80.^62^ The amount of goods that bastards passed to their lords after death and the number of mortmain payments increased substantially in the cities of Sluis, Courtrai, and Bruges in the years 1400–1, as well as in smaller towns such as Geraardsbergen and Aalst.^63^ Serious plagues continued intermittently throughout the fifteenth century too. A significant outbreak struck in the late 1430s in the Southern Netherlands (though, as noted later, coupled with famine conditions), where documents that recorded possessions coming into the hands of the duke show that one in three bastards died in Bruges at this time.^64^ This plague period also caused exceptionally high mortality in Tournai and Arras: with the number of new wills rising to 328 in 1438 (from an annual average of close to 50) in the first case,^65^ and the burial of 802 bodies in 1438–9 in the second case—a significant leap from the 46 pits dug when the situation normalized in the 1440s.^66^

Our newly compiled database of mortmain accounts further validates the above findings. It provides an excellent source of mortality information from the county of Hainaut for the Black Death and subsequent plagues (25,610 individuals in total for the period 1349–1450).^67^ Mortmain— a death fee on possessions, paid to the count by a large subsection of the population—should not be confused with the same legal term used in regions with English, French, or Anglo‐Norman juridical authorities, where it instead refers to the alienation of land to the dead hand of the Church.^68^ The Hainaut mortmain instead is closer to the English heriot, since both were death duties usually paid in the form of the best live beast or chattel of the deceased. The main difference between the mortmain and the heriot is that heriots were levied on tenants and payable to a manorial lord, while mortmain was levied on a broader range of people who came from an ancestral lineage of servitude, but now enjoyed free status.^69^ While heriots targeted a specific socio‐economic group and only affected heads of households, mortmain in Hainaut included all men and women who had reached the age of majority, plus some emancipated children in a small number of cases.^70^ As table 1 shows, women made up a significant proportion of the total database in the period 1349–1450 (43.7 per cent of the entire database). The average sex ratio was just 1.07:1 when we exclude localities with special local stipulations excluding women.^71^ Table 1 also shows that the mortmain sometimes recorded more than one person from the same household: 2.5 per cent of the database were husbands and wives together and 0.7 per cent were siblings. Three groups were exempt: (a) ‘*les gens d'origine franche*’ (a form of noble status transferred through maternal lineage), (b) ‘*les seigneurs haut‐justiciers*’ (noble judges), and (c) religious professionals.^72^ While certain people of high social status were excluded, it did not exclude all high‐ranked individuals, as the accounts sporadically record, for example, ‘*sire*’ and ‘mayor of *x*’. Mortmain also extended to the poorest in society, even beggars. The wide disparities in the value of the goods impounded as death duty indicate that the source does not represent a narrow subset of society.

**Table 1:** Distribution of mortmain entries, Hainaut, 1349–1450

	Total database		Total without localities with special stipulations	
	%	N	%	N
Male	54.4	13,940	50.8	11,292
Female	43.7	11,200	47.5	10,559
Unknown	1.8	470	1.8	396
Total	n.a.	25,610	n.a.	22,247
Sex ratio	1.16		1.07	
Husband–wife entries	2.5	636	n.a.	n.a.
Sibling entries	0.7	195	n.a.	n.a.

*Sources*: ADN, B 12122–12226; ARB, I, 004, 17867–73.

The Hainaut mortmain accounts do, of course, have limitations—for example, delayed or incomplete registration during political upheaval or military activity—yet they allow us to compare deaths in crisis years (that is, plague years) with those in ‘normal’ years.^73^ The source has been used before, notably by Blockmans, but his work was unfortunately based on a dataset from an MA thesis written in the 1950s which contained errors—sometimes recording double entries, and sometimes failing to record more than one person in a line of the document (unsurprising given the erroneous view that the mortmain only recorded heads of household).^74^ Sivéry also used mortmain accounts; however, he did not incorporate the accounts for 1350–1, which were kept not in Lille but in the Brussels National Archives.^75^ Similarly, in a subsequent article on the 1400–1 plague outbreak, he did not use accounts for September 1400 to September 1401, again kept in Brussels.^76^ As a result, these studies understate the severity of plagues, and this current study is the only one to use all available mortmain documents for the Black Death and the plague of 1400–1.

One further methodological issue is that the number of deaths appearing in mortmain accounts was not a direct indicator of death, but could also increase or decrease, depending on the rights of the overlord to take goods after death. For nearby Flanders, for example, Kittell argued that the count of Flanders was able to subject more people to mortmain payment over the second half of the fourteenth century, exploiting a population weakened by persistent plagues and upsurges in conflict.^77^ Kittell based her evidence on the changing structure and terminology used in the Flanders mortmain accounts, which were only standardized from the 1370s onwards. This was not the case for Hainaut, however, as the accounts there were standardized from the very first extant records of 1349, with terminology and structure remaining unchanged until after 1500. In Hainaut, subjection to mortmain was passed on through maternal family lineage, and thus the count could not arbitrarily subject more people at random.^78^

**Figure 1:**
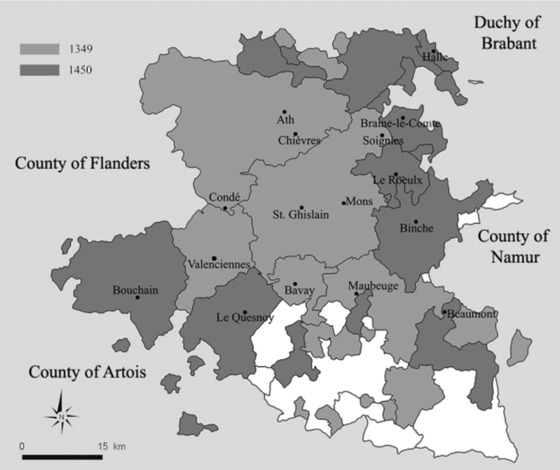
County of Hainaut with extent of territorial reach of mortmain in 1349 and 1450

**Figure 2:**
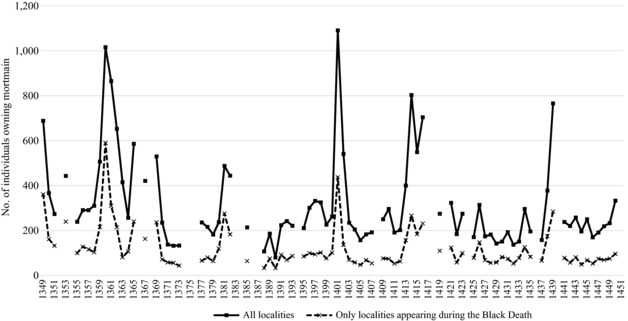
Annual mortality based on individuals owing mortmain, Hainaut, 1349–1450, adjusted for missing months in a year *Sources*: ADN, B 12122–12226; ARB, I, 004, 17867–73.

A more serious issue is that the number of districts (and thus localities) included in the account fluctuated over time, which was probably caused by expansions and contractions in jurisdictional grip over territory.^79^ The map in figure 1 shows the difference between the districts included in 1349 and the larger number included in 1450. Since the number of localities included for the Black Death was relatively low compared to later years, the mortmain provides a minimum impression of plague mortality. The effect of the omitted localities can be demonstrated by comparing trends in figure 2. In figure 2 we can see the total number of people per year paying mortmain (and therefore the number of dead) in Hainaut from 1349 to 1450. Although a mortality spike can be seen in the years 1349–51, the simple total gives the impression that the Black Death was much less substantial than many of the plagues that were to follow. While this could be evidence that the Black Death failed to touch many localities, the more likely reason is that the territorial reach of administering the record was more restricted. Rather than individual localities, whole districts are missing in the earlier records, including Braine‐le‐Comte, Binche, Halle, Beaumont, and, to a large extent, Valenciennes. One way of dealing with this problem is to include only deaths in localities that are present in the accounts during the Black Death and for the majority of the years to 1450.^80^ In the adjusted total in figure 2, the Black Death spike is much closer to the other plague spikes, although it is still lower than in the crises of 1360 and 1401. It must be noted that since the first account starting in June 1349 is in poor physical condition with parts missing or unreadable, approximately 20 per cent of the content is lost. Furthermore, the first account only covers 9.5 months rather than a whole year. Accordingly, figure 2 shows mortmain mortality in 1349 using a multiplier of 1.25 (to cater for the missing 20 per cent of information) and 1.26 (to cater for the missing months). The issue of missing manuscript information does not affect any other year up to 1450, although we have used multipliers for all other years where the account was not equivalent to or exceeded a full year.^81^

**Figure 3:**
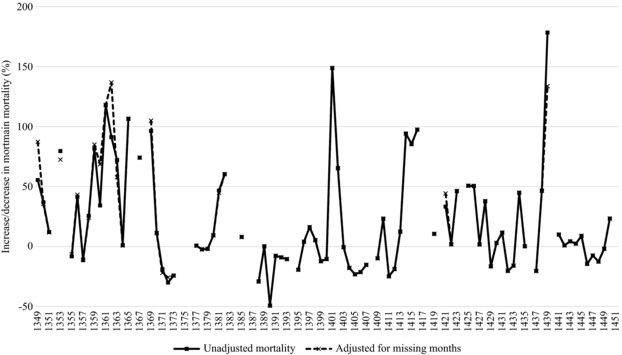
Annual mortality based on increase or decrease (%) from the average number of mortmain per locality across the period 1349–1450, Hainaut *Sources*: ADN, B 12122–12226; ARB, I, 004, 17867–73.

Another approach to plague mortality is to calculate the relative annual percentage change in mortmain against long‐term average mortmain figures for the whole period 1349–1450. We calculate for each locality in every year (for which mortmain data are available) the increase or decrease in mortmain deaths as a percentage of that locality's average mortmain deaths for the whole period 1349–1450.^82^ An overall average of these local measures is then calculated for every year. Using this methodology, figure 3 reveals a significant Black Death mortality spike, particularly when adjusting for missing months and the physical damage to the manuscript. However, this is still somewhat understated, since larger settlements were hit harder during the Black Death according to the mortmain data (as we show later on in table 2), yet this method weights all localities equally regardless of their size.

**Table 2:** Urban–rural mortality ratios in plague years, Hainaut, 1349–1450

Urban–rural mortality summary			
	Urban (n)	Rural (n)	Urban–rural ratio
All years 1349–1450	8,385	17,225	0.49
Plague years	4,238	7,913	0.54
Non‐plague years	4,147	9,312	0.45
p‐value	≤0.0001		

Urban–rural mortality for individual plague years					
Plague year	Urban–rural ratio	Plague year	Urban–rural ratio	Plague year	Urban–rural ratio
1349	0.92	1363	0.80	1413	0.65
1350	0.52	1369	0.32	1414	0.50
1351	0.72	1380	0.85	1415	0.32
1358	0.51	1381	1.05	1416	0.43
1359	0.36	1382	0.28	1425	1.25
1360	1.10	1400	0.97	1426	0.34
1361	0.41	1401	0.70	1438	0.53
1362	0.16	1402	0.51	1439	0.44

*Note*: p‐value calculated through a Chi‐squared test; it is from a comparison of the ‘Urban’ and ‘Rural’ categories and between the ‘Plague years’ and ‘Non‐plague years’ groups, and is highly significant.

*Sources*: ADN, B 12122–12226; ARB, I, 004, 17867–73.

Ultimately, although showing a more severe impact of the Black Death than hitherto believed, even our revised presentation of the Hainaut mortmain data suggests that mortality was lower during the Black Death than later epidemics. However, there are good reasons to believe that the Hainaut figures understate the scale of mortality during the Black Death. Some parts of Europe saw raised mortality as early as 1347–8, but the first mortmain account only survives from June 1349, coinciding with the arrival of the Black Death in the city of Valenciennes.^83^ It seems likely that the first extant account, starting on 24 June 1349, was created as a direct consequence of hyper‐mortality, and thus misses the initial impact of the Black Death in the region. Counts of deaths in the mortmain during 1348–50 should be considered a lower‐bound estimate of mortality.

This is further supported by clear evidence that the administration of mortmain was disrupted during the Black Death. In the accounts for 1350–1 we find a second report of additional deaths for certain localities made by officials other than the original bailiff. This is not encountered in any other document over the following century.^84^ In all likelihood, this is due to a change in the local bailiff after the previous incumbent had died or fled—indicative of severe mortality, but also of administrative disruption that may have led to an under‐reporting of deaths. In fact, the local administration was probably caught off‐guard, since the accounts were probably triggered by the sudden hyper‐mortality, disrupting earlier administrative procedures. As plague became a recurring disease, it is likely that the administration found ways to record deaths to a much fuller extent, but the Black Death was a shock without precedent, disrupting the laborious process of selecting and collecting the impounded object, finding a buyer, haggling over its value, auctioning it, and recording the money received.^85^ The spike seen in 1353, for example, may simply reflect delayed recording of the mortmain, and therefore be testament to the administrative disruption caused by the Black Death. Disruption is also reflected in the abnormally high ratio of deaths of siblings to deaths of husband–wife couples in 1349. Sibling deaths were typically lower than husband and wife deaths during plague years (from 0.13:1 to 0.6:1, except during 1380–1 when the ratio rises to 1:1 and 1.5:1 respectively), and were never high in non‐plague years, yet in 1349 they were 3.67 times more common than husband–wife deaths, much higher than other plague years. Husband and wife deaths were probably under‐reported because it was difficult to identify cases in which whole households perished.

Finally, direct comparison between the Black Death and later mortality spikes has to contend with the issue that plague may not have been the sole driver of raised mortality in later years. For example, the mortality spike of 1439 in Hainaut occurred during an extremely cold decade—one of the coldest of the past millennium,^86^ leading to a period of famine.^87^ Recent research on Dijon has suggested that raised mortality in 1438–40 may in fact have a ‘different, possibly waterborne, disease involved’ based on the spatial concentration of mortality.^88^

Whatever the size of these effects on our estimate for 1349–51, evidence for extremely severe mortality shocks in 1360 or 1400 cannot be used to infer a ‘weak’ impact of the Black Death. In fact, what the mortmain records demonstrate is that rather than a ‘light touch’ of the plague in the Southern Netherlands, the severe outbreak of the Black Death was followed by recurrent serious plagues throughout the fourteenth and fifteenth centuries. The Southern Netherlands faced a harsh late medieval epidemiological regime, not a light one: something that fits much better with our understanding of the region as highly urbanized, densely populated, highly commercialized, and mobile.

The uncertainty over the causes of raised mortality in 1439 leads us to consider a final issue: can we identify a spike in the number of mortmain, or coffins produced, or wills written, as caused by plague? Thoen and Devos argue that due to the paucity of sources, it is not possible to separate late medieval plague from other diseases and causes of mortality.^89^ Currently we lack studies identifying the pathogen in late medieval plague outbreaks other than the Black Death (an exception is the identification of *Yersinia pestis* at Bergen‐op‐Zoom during ‘*pestis secunda*’).^90^ To address this, we have compared the timing of the major mortality spikes in the Hainaut mortmain database with a database that identifies primary and secondary documentary sources making explicit or indirect reference to plague in the Low Countries between 1348 and 1499. A similar enterprise was undertaken by Biraben, but, while particularly thorough for France, his coverage of the Low Countries was unsystematic.^91^

Naturally, caution should be exercised with this kind of data. Many works that refer to plague do not provide unequivocal evidence, but point to a disease of some kind or a mortality crisis that *may* have been caused by plague. The sources only begin to differentiate explicitly between diseases from the second half of the fifteenth century: good examples are the references to epidemics in the Duchy of Guelders in 1472–3 and 1497–8 as ‘*rode loop*’ and ‘*rood melisoen*’ (dysentery) and ‘*pokken*’ (smallpox) respectively.^92^ In Tielt in 1492–3, ‘*‘t rood mimzoen*’ (dysentery) is mentioned,^93^ and a similar example of ‘*louppenden buyck*’ (diarrhoea) can be found in 1473;^94^ both occurred when many other places in the Low Countries were experiencing plague, indicating that many diseases could also be raging simultaneously. A broader comparative view helps: some sources mention a ‘*haestige sieckte*’ (rapid sickness), which on its own is vague, if it were not for specific mentions of plague in the same year elsewhere.^95^ We categorize references to plague in three ways: quantifiably demonstrable increases in mortality; descriptive mentions of plague in contemporary sources; and descriptive mentions of plague in later sources (for example, chronicles after the epidemic). To be included in our analysis, secondary sources must state where they found the evidence or at least what the evidence represents.^96^

Matching the Hainaut mortality spikes with mentions of plague in medieval Low Countries sources suggests that plague outbreaks occurred in 1349–51, 1358–63, 1368–9, 1380–2, 1400–2, 1413–16, 1425–6, and 1438–9, even if they were potentially coupled with other afflictions, as seen in 1438–9. For 1353, 1364–7, 1390, 1419, 1423, and 1434, there was no documentary evidence of plague, although the disease cannot be ruled out.

## III

In previous literature, most information on plague mortality in the Low Countries has been taken from urban evidence, while the impact of the Black Death in the countryside has been of peripheral interest.^97^ This is problematic on three grounds. First, it is widely accepted that the initial Black Death across Europe affected both city and countryside to a severe degree.^98^ Second, recent burial register evidence for the early modern period has shown that the countryside of the Low Countries often experienced severe plague epidemics indicated by high rural mortality rates.^99^ Third, while previous literature definitively asserted that the Black Death and subsequent plagues of the Second Pandemic were caused by flea bites from infected rats, now there is significant unresolved debate about the nature of the precise vectors and modes of transmission involved.^100^ Recent literature highlights plague's capacity to kill people in close confinement,^101^ and therefore suggests the potential for human‐to‐human transmission, whether through a vector (human flea or louse) or even directly.^102^ Whatever the reality, the reduced emphasis on the rat as *sole* vector^103^ also legitimizes the view that plague was capable of spreading into rural, even isolated, environments—perhaps even originating in rural environments from a prior enzootic phase, and set off by certain climatic or ecological changes.^104^ In the Southern Netherlands, the likelihood of plague spreading to (or from) the countryside was high, given that distances between villages and towns were small, rural population density was high, and movement and interaction between urban and rural people was easily facilitated in a commercialized environment that was also subject to frequent military conflict.

Nicholas was one of the first scholars to find an explicit reference to rural plague mortality during the Black Death. The *Acta Capituli Sancti Donatiani* (Chapter Act‐Books of St Donatian's Cathedral), from 30 September 1350, referred to exceptionally low tithe revenue.^105^ As Vermeersch has shown, further systematic use of the source can demonstrate abnormally high rural mortality. The canons played an important role in appointing parish priests in Bruges and its surrounding rural hinterlands, and the *Acta Capituli* of 1349 contains appointments for rural parishes left vacant. In September 1349, a new parish priest was appointed for numerous villages, including Sint‐Kruis, Uitkerke, Loppem, Sint Willibrord, Middelkerke, Blankenberge, Dudzele, and Sint‐Jacobskapelle, while in Diksmuide both the parish priest and chaplain of the Beguine chapel died. In October 1349, the parish church of Dudzele was in need of a new priest again, just one month after installing the previous incumbent. At the end of September 1349, the demand for new priests was so high that there were too few to fill the vacancies.^106^ The accounts of the *Officie van den Brode en van de Foraniteit* (an institution responsible for providing food and shelter for inhabitants of the chapter) for the years 1348 and 1352 are another indicator for rural mortality in the areas around Bruges. This charitable institution was funded by *cijnsrechten* (a form of tithe) on property in Bruges and its rural hinterlands. Of the 13 tithe holders in 1348 from the villages of Uitkerke, Varsenare, Houthave, Moerkerke, and Zuienkerke, only nine remained in 1352. The complete account including parishes in Bruges shows that 28 out of 80 tithe holders disappeared between the same years.^107^

Two rural villages in Walloon Flanders that unusually have household counts for just before and just after the Black Death experienced contractions of 25 per cent and 18 per cent between 1347 and 1351.^108^ Such a decline is corroborated for a few select Hainaut villages that were also able to give pre‐Black Death hearth figures. The three villages of Pont‐sur‐Sambre, Forest‐en‐Cambrésis, and Louvignies‐Quesnoy lost 27 per cent, 60 per cent, and 58 per cent of their hearths, respectively, between 1286 and 1365,^109^ even if we concede that this gap between dates includes the famine of 1315–17 and the second plague outbreak of 1358–63, and take into account the methodological challenges regarding hearth counts already described above. In other source material, the mortality rate for farmers in the surrounding rural hinterlands of Cambrai, as tenants of the chaplains of Notre Dame Cathedral, was more than a third in the mid‐fourteenth century.^110^

Plague outbreaks continued to affect the countryside in intermittent stages throughout the fourteenth and fifteenth centuries too. Previous literature has downplayed the rural spread of the outbreaks in many parts of Europe after the Black Death; Naphy and Spicer state, for example, that ‘the second outbreak and subsequent plagues differed [from the Black Death] in that it was more confined to urban areas’ and ‘subsequent outbreaks tended to be localised in population centers’.^111^ The Hainaut mortmain database does not support this view for the Black Death, however, since the urban–rural ratio of mortmain mortality was high during certain years of the Black Death.^112^ The figure of 0.92:1 in 1349, for example, was the fifth‐highest out of all years from 1349 to 1450, and was much higher than the average urban–rural ratio for all years (0.49:1) calculated in table 2.^113^

For the later plagues, the Hainaut data partially support the view that plague mainly affected towns. For example, the only time in the period 1349–1450 when the urban–rural mortality ratio rose above 1:1 in absolute numbers was during the plagues of 1360, 1380, and 1425, and table 2 shows that the urban–rural ratio in mortmain deaths was generally higher during plagues than in non‐plague years (0.54:1 compared to 0.45:1). However, there was not a simple chronological development from more universal to more territorially restricted outbreaks. Table 2 shows that in several plague years, such as 1361–2, 1369, and 1415–16, mortality was relatively more severe in rural areas than in cities, compared to the norm. Indeed, when employing the same methodology already used for figure 3, where mortmain figures are calculated as a percentage increase or decrease in the average for the whole period 1349–1450, table 3 shows that many recurring plagues were territorially pervasive.^114^ That is to say, high proportions of rural localities during plague periods were displaying increases in mortmain that were 50 per cent higher than their 1349–1450 average.

**Table 3:** Share of localities with raised mortality during plague years in Hainaut, 1349–1450

	Urban		Rural	
Plague years	%	n	%	n
1349–51	50	2	55.7	39
1358–63	70	7	75.5	80
1368–9	62.5	5	54	27
1380–2	55.6	5	45	36
1400–2	90.9	10	72	72
1413–16	83.3	10	80.7	88
1425–6	50	4	37	20
1438–9	77.8	7	74.7	59

*Sources*: ADN, B 12122–12226; ARB, I, 004, 17867–73.

Other sources also show that later plagues spread into the countryside in the Southern Netherlands. Rural tenant farmers of Notre Dame Cathedral in Cambrai died in significant numbers in the outbreaks of 1360–2 and 1368–9: roughly 33 per cent of those listed in 1360–2 (equivalent to the death rate among the chaplains resident in the city), and 25 per cent of those listed in 1368–9 (exceeding the 10 per cent death rate for the cathedral chaplains).^115^ In the rural Land van Waas during the outbreak of 1368–9, domain accounts of the castle of Beveren show the death of 12 servants, when the usual number in the previous and following years was three to six.^116^ In the village of Petegem in inland Flanders, there was a clear peak in the receipt of death duties during the 1368–9 outbreak, increasing to 13 between June 1368 and June 1369, from an average of four over the previous and following years.^117^ The village of Beuvry in Artois in the plague year of 1368 saw its number of ‘reliefs’ shoot up to 163, a substantial increase from a normal annual average of around eight, and, according to Derville, 40 per cent of the adult population died in 166 days.^118^

## IV

We argue that the Black Death was more severe in the Low Countries, and particularly the Southern Netherlands, than previously accounted for. Serious plagues continued to occur throughout the rest of the late middle ages, establishing a harsh plague regime. These plagues were sometimes territorially pervasive, affecting the countryside.^119^ The question then arises as to why we have established a different view from that entrenched within the historiography of the ‘light touch’ of plague in the Low Countries.

Part of the reason for the ‘light touch’ view perpetuating itself over such a long time is connected to the limitations of the sources mentioned in section I. However, the contrast between the historiography and our view also stems from previous overemphasis on cities and particular types of source information. The problem is that mortality crises become less traceable from urban population data since cities had a distinct and different demographic regime to that of the countryside. It is widely accepted now that serious epidemic diseases causing mortality shocks in the late medieval and early modern period decimated cities, but these losses were frequently exhibited over the short term. Urban populations replenished themselves through inward migration from the rural hinterlands.^120^ Accordingly, some scholars have used stable or even rising urban population figures as evidence for the weak impact of the plague,^121^ yet this may be an index for rapid and full rural–urban migration that hides the mortality effect.^122^

Although migration is hard to document empirically for the middle ages, there are also some signs that the Black Death helped stimulate rural–urban migration in the Southern Netherlands. One agreement drawn up between the coastal town of Nieuwpoort and Furnes‐Ambacht on 3 April 1350 explicitly mentions that recent plague mortality had caused inhabitants to move from the villages of Leke and Klerken to the town.^123^ In Bruges, the urban government recognized this and in the wake of the Black Death imposed higher entrance fees (*poortersgeld*), especially for ‘foreigners’ or non‐Flemings.^124^ This acted as only a moderate deterrent for immigration since migrants did not need to become burghers simply to live in the city.^125^ Yet despite understating rural–urban migration, registrations of new burghers do reveal mass mobility in the wake of the Black Death: in Lille the number of new burghers in the years 1350–1 exceeded 200 (the overwhelming majority through purchase rather than birth), and yet only on six other occasions between 1327 and 1370 did the number ever go above 100, and in no other year did it exceed 150.^126^ There were a number of conditions within the Southern Netherlands that facilitated post‐plague rural–urban migration. By the time of the Black Death, commercial connections between city and countryside were already strong, as market‐orientated peasants from inland Flanders, for example, came to the cities to sell their surpluses.^127^ Furthermore, there were few institutional impediments to the migration of people towards the cities in the mid‐to‐late fourteenth century, at least not from the rural side. While in some parts of Europe, manorial or seigneurial lords tried to keep their tenants rooted to their localities (often without success), leading in some places to rather extreme labour legislation,^128^ the weaker grip of feudal coercion in the Southern Netherlands by the fourteenth century paved the way for freer movement of rural people.^129^ Finally, even with plague mortality in the countryside, there were enough rural inhabitants prior to the Black Death to serve as a reservoir for urban replenishment. The countryside in inland Flanders, for example, was perhaps the most densely populated in the whole of western Europe, perhaps rivalled only by the *contadi* of the Tuscan towns.^130^

## V

Although the fanciful notion that the urbanized and commercialized areas of the Southern Netherlands managed to avoid the worst excesses of the plague is no longer maintained, the literature has still downplayed its overall severity in the late medieval Low Countries, particularly for the initial Black Death. The perpetuation of the story of the ‘light touch’ of the plague in the Low Countries has not just gained general acceptance,^131^ but has in fact been a component of some of the most important recent explanations for the social and economic efflorescence of parts of the Low Countries in the transition from the late middle ages to the early modern period, and the relative ease with which it recovered its population, even if this is viewed as working in tandem with other endogenous processes.^132^ Our re‐evaluation of the basic empirical ‘fact’ of a ‘light touch’ of plague in the Low Countries, and the Southern Netherlands in particular, has indirect consequences for how we explain the rapid and full recovery of the population. In fact, this overall recovery may have been overstated, with the true picture being obscured by resilient urban settlements taking in migrants from increasingly decimated rural areas: the Low Countries did not necessarily recover its population to a greater degree than elsewhere; it experienced a greater degree of rural–urban migration. Yet even if the rapid and full demographic recovery story is correct, this was most likely connected to the endogenous processes of recovery itself, rather than the chance ‘good fortune’ of experiencing weaker exogenous biological shocks. Instead of reduced mortality, one explanation may be a high fertility regime: the consequence of high real wages and low ages of marriage in the post‐Black Death era.^133^

[Correction added on 28 May 2018, after first online publication: Gilliodts‐van Severen has been deleted from the references list.]

## Supporting information

S1. Plague mentions in the Low Countries sources, 1349–1499

S2. Hainaut mortmain mortality data, 1349–1450

## Footnotes

1
van Werveke, *De Zwarte Dood*; idem, ‘Nogmaals: de Zwarte Dood’. By the ‘Black Death’ we refer to the first mid‐fourteenth‐century outbreak of plague, to distinguish it from other plagues of the Second Pandemic; see Alfani and Murphy, ‘Plague’, p. 315.

2 For the south: Maréchal, ‘De Zwarte Dood’; Blockmans, ‘Social and economic effects’; Vermeersch, ‘De Zwarte Dood’. For the north: de Boer, *Graaf en grafiek*; Noordegraaf and Valk, *De Gave Gods*; Ladan, *Gezondheidzorg*, ch. 2; Hoppenbrouwers, *Een middeleeuwse samenleving*, pp. 58–60. [Correction added on 28 May 2018, after first online publication: Inclusion of publication Vermeersch, ‘De Zwarte Dood’ after Blockmans.]

3 For an overview, see the sections on England, Italy, and France in Benedictow, *Black Death, 1346–1353*.

4
Hatcher, ‘Mortality’; Hatcher, Piper, and Stone, ‘Monastic mortality’; Nightingale, ‘Some new evidence’; Alfani, Carbone, del Bo, and Rao, eds., *La popolazione italiana*.

5
Malanima, ‘Economic consequences’, p. 314.

6
Carpentier, ‘Autour de la peste noire’, p. 1070.

7 Reproduced, for example, in an introduction in Slack, *Plague*, p. 19. Subsequent reproductions have been criticized in Mengel, ‘Plague on Bohemia?’. See also the absence of plagues in the Low Countries in recent reproductions of the Jean‐Noël Biraben data: Büntgen, Ginzler, Esper, Tegel, and McMichael, ‘Digitizing historical plague’, p. 1587; Schmid, Büntgen, Easterday, Ginzler, Walløe, Bramanti, and Stenseth, ‘Climate‐driven introduction’; Yue, Lee, and Wu, ‘Navigable rivers’; eidem, ‘Trade routes’. Criticized in Roosen and Curtis, ‘Dangers’.

8
Cohn, *Black Death*, pp. 179–80; Scott and Duncan, *Biology of plagues*, p. 87.

9 Our translation from Blockmans, Pieters, Prevenier, and van Schaïk, ‘Tussen crisis en welvaart’, p. 57. An estimated death rate of 16–25% for Flanders during the Black Death can be found in Nicholas, *Medieval Flanders*, p. 266.

10 Christakos, Olea, Serre, Yu, and Wang, *Interdisciplinary public health reasoning*, p. 222.

11
Aubry, ‘Les mortalités lilloises’, p. 336.

12
Thoen and Devos, ‘Pest’; Derville, ‘La population’; Helin, ‘Les Pays‐Bas’; Thoen, ‘Historical demography’; Sivéry, ‘La Peste Noire’; idem, *Structures agraires*, pp. 610–11.

13
Sivéry, ‘La Hainaut’; Thoen *Landbouwekonomie*; Despy, ‘La “Grand Peste Noire”’.

14
Thoen and Soens, ‘Family or the farm’, pp. 197–8.

15
van Bavel, *Manors and markets*, pp. 279–80.

16
van Bavel, ‘Medieval origins’, p. 67.

17 Importantly, it has been noted that there were strong regional divergences; van Bavel, ‘People and land’. For example, the population of inland Flanders is said to have recovered more quickly than coastal Flanders; Thoen and Soens, ‘Family or the farm’.

18
Malanima, *Pre‐modern European economy*; Broadberry, Campbell, Klein, Overton, and van Leeuwen, *British economic growth*.

19
Pounds, ‘Population’, p. 379.

20
Pamuk, ‘Black Death’, p. 308.

21
van Bavel and van Zanden, ‘Jump‐start’, p. 515. The connection between a mild plague shock and favourable economic and demographic growth is further asserted in van Zanden, ‘Third road to capitalism?’, p. 90; Hoppenbrouwers, ‘Van waterland tot stedenland’, p. 136.

22
Thoen and Devos, ‘Pest’, p. 115.

23
Dhérent, ‘Abondance et crises’, p. 366.

24
Slicher van Bath, *Agrarian history*, pp. 89–90.

25 This is asserted *passim* in most of the essays in Alfani and Ó Gráda, eds., *Famine*. One connection between famine and plague may have been social disruption and breakdown rather than nutritional deficiency; Alfani, *Calamities*, p. 45.

26
Jordan, *Great Famine*. However, some argue that greater exposure to the Great Famine may have removed the frailest individuals, creating a cohort less likely to die from the Black Death; DeWitte and Slavin, ‘Between famine and death’, p. 47.

27
Alfani and Murphy, ‘Plague’, p. 322.

28
Kowaleski, ‘Medieval people’, p. 577.

29 As noted in Klapisch‐Zuber, ‘Plague’, p. 131. Recent work has tried to limit these problems by using archaeological indicators over larger areas instead; Lewis, ‘Disaster recovery’.

30
Thoen, ‘Historical demography’.

31
Derville, ‘Le nombre des habitants’; idem, ‘La population’; Bocquet, *Recherches*; Arnould, *Les dénombrements de foyers*; Cuvelier, *Les dénombrements de foyers*; van Schaïk, *Belasting*. Other forms of early censuses (with large chronological gaps) have been used for areas of late medieval North Holland; see de Boer, *Graaf en grafiek*; Lesger, *Hoorn*.

32 For the early modern period, see Curtis, ‘Was plague an exclusively urban phenomenon?’; Cohn and Alfani, ‘Households’; Whittles and Didelot, ‘Epidemiological analysis’. For the late middle ages, see Curtis and Roosen, ‘Sex‐selective impact’.

33
Noordegraaf and Valk, *De Gave Gods*.

34
Derville, ‘La population’, p. 530.

35
Rommes, ‘Plague’.

36
Blockmans, ‘Social and economic effects’, p. 839; de Boer, *Graaf en grafiek*, p. 32; Nicholas, *Town and countryside*, pp. 230–3.

37
Deregnaucourt, ‘Autour de la mort’, vol. I, p. 60. Evidence used in Cohn, *Black Death*.

38 This links to Cohn's argument that important changes to testamentary recording occurred in the wake of ‘*pestis secunda*’ as a traumatic reliving of the previous mortality shock of the initial Black Death; Cohn, ‘Renaissance attachment to things’. Benedictow also asserts that wills and testaments are better seen as indicators of attitudes to death rather than mortality outright; Benedictow, *Black Death, 1346–1353*, pp. 268–9.

39 Our database calculated from Wynant, ed., *Regesten*, vol. I, pp. XVIII–XXI; Stadsarchief Gent, Oud Archief, no. 330 (1349–1440). Although probate inventories were recorded from 1330, these are the earliest extant records.

40
Rogghé, ‘De Zwarte Dood’, p. 836.

41
Vermeersch, ‘De Zwarte Dood’, p. 14.

42
DeWitte, ‘Archaeological evidence’, p. 65.

43
Maréchal, ‘De Zwarte Dood’, p. 379.

44
Vandeburie, ‘De Zwarte Dood’, p. 299.

45
Maréchal, *De sociale en politieke gebondenheid*.

46
Vandeburie, ‘De Zwarte Dood’, p. 278.

47
Blockmans, ‘Social and economic effects ’, p. 839.

48 Vermeersch, ‘De Zwarte Dood’, p. 15. [Correction added on 28 May 2018, after first online publication: “Vermeesch” has been corrected to “Vermeersch” in the main text and the Footnote 48.]

49
Vandeburie, ‘De Zwarte Dood’.

50
Derville, ‘La population’, p. 524.

51 Ibid., pp. 523–5.

52 Archives Départementales du Nord, Lille (hereafter ADN), Lille B, no. 10817.

53 Archives Municipales Saint‐Omer, Registre au Renouvellement de la Loi, no. 5.

54
Derville, ‘La population’, pp. 523–5.

55 Bled, Haigneré, and Dewitte, eds., *Les chartes de Saint‐Bertin*, vol. II, no. 1710.

56
Derville, ‘Le nombre d'habitants’, pp. 281–2.

57
Aubry, ‘Les mortalités lilloises’, p. 333.

58 Stadsarchief Gent, Archief H. Geesttafel van St. Niklaaskerk, no. 30.

59 Archives Départementales du Pas‐de‐Calais, Arras (hereafter ADPdC), Série H‐Dépôt: Archives hospitalières déposées (1167–1968), E10, no. 1.

60 Ibid., E13, no. 1.

61
Decamps, ‘La maladie contagieuse’, p. 139.

62
Vandenbroeck, *Extraits analytiques*, vol. I, pp. 47–8.

63
Vangassen, ‘De pestepidemieën’, pp. 29–32.

64 Ibid., pp. 61–70.

65 Ibid., p. 71.

66 ADPdC, Série H‐Dépôt: Archives hospitalières déposées (1167–1968), E1, nos. 16–20.

67 Mortmain accounts from ADN, B 12122–12226; Algemeen Rijksarchief Brussel (hereafter ARB), I, 004, 17867–73. The raw data can be accessed in online app. S2.

68
Kittell, ‘Death’, p. 192.

69
Verriest, *Le servage*, pp. 248–50.

70
Benedictow, *Black Death, 1346–1353*, p. 375; Arthur, ‘Black Death’, p. 60. Previous scholars working on the Hainaut mortmain record assumed it was only for heads of household too; Sivéry, ‘La Hainaut’, p. 435; Blockmans, ‘Social and economic effects’, p. 937.

71 We remove the cities of Maubeuge and Bavay where women supporting a child did not pay mortmain; Verriest, *Le servage*, p. 309. Rural localities with a level of female mortmain exclusion were Froidchapelle‐Fourbechies, Rousies, Montbliart, Solre‐Saint‐Géry, Villers, Vaulx, Leval‐sous‐Beaumont, Acren‐Saint‐Martin, Rance, Taisnieres, and Vieux‐Condé.

72 Ibid., pp. 312–22.

73
Thoen and Devos, ‘Pest’, p. 28.

74
Blockmans, ‘Social and economic effects’, pp. 850, 856.

75
Sivéry, ‘La Hainaut’. The 1350–1 account is also omitted in Blockmans, ‘Social and economic effects’.

76
Sivéry, ‘La Peste Noire’.

77
Kittell, ‘Death’, p. 192.

78
Verriest, *Le servage*, pp. 253–66.

79 Certain districts and localities were sometimes leased out by the Count, explaining why they returned to the Count's portfolio some years later. Other lay and ecclesiastical lords also sometimes acquired mortmain rights through alienation and purchase; ibid., p. 289.

80 This series includes the cities of Ath, Bavay, Maubeuge, Mons, and St‐Ghislain, and the villages of Crespin, Cuesmes, Herchies, Horrues, Jemappes, Jurbise, Kieuraing, Lens, Masnuy St Jean and St Pierre, Montignies, Neufville, Quaregnon, St‐Symphonien, Hon, Hyon, Cambron St‐Vincent, and Mainvault. Localities included had to appear during the Black Death and be present for at least 75% of the following years. For rural localities this allows for years when absences simply occur because no mortmain payments were made (many rural localities recorded only one or two names in ‘normal’ years).

81 Not all accounts extend neatly from 1 Jan. to 31 Dec., but in shortened periods which we combine to make up a year. In the period 1349–99, there were 18 occasions when the combined accounts did not make up an exact year, and in the period 1400–50, there were two occasions. Usually this was only an increase or decrease of a month or less from a full year.

82 We would have preferred to calculate a percentage change from the ‘previous years'’ rate, as used for burial records in Alfani, ‘Plague’; followed in Curtis, ‘Was plague an exclusively urban phenomenon?’. However, since the dataset only starts in 1349, it is not possible to calculate a ‘previous years'’ figure for the Black Death period.

83
Sivéry, ‘La Hainaut’, p. 438.

84 ARB, I, 004, no. 17867.

85
Kittell, ‘Death’, p. 191.

86
Camenisch, ‘Endless cold’. For the 1430s in a broader European perspective, see Camenisch et al., ‘1430s’.

87
Derville, *L'agriculture du Nord*, p. 215; Verhulst, ‘L’économie rurale’, p. 70; Blockmans, ‘Privaat en openbaar domein’, p. 711.

88 Galanaud, Galanaud, and Giraudoux, ‘Historical epidemics’.

89
Thoen and Devos, ‘Pest’, pp. 20–1.

90 ‘*Pestis secunda*’ refers to the second plague outbreak of the Second Pandemic that followed the Black Death. Green and Schmid, ‘Plague dialogues’; previously (erroneously) identified for the Black Death outbreak: Haensch, Bianucci, Signoli, Rajerison, Schultz, Kacki, Vermunt, Weston, Hurst, Achtman, Carniel, and Bramanti, ‘Distinct clones’. On laboratory studies with *Yersinia pestis* findings for the Black Death, see Little, ‘Plague historians’; Benedictow, *Black Death and later plague epidemics*, pp. 81–2.

91
Biraben, *Les hommes*. Noordegraaf and Valk rectified this by creating a database of their own, which, although thorough and citing original sources, began from the year 1450 and was focused on Holland; see Noordegraaf and Valk, *De Gave Gods*, app.

92
van Schaïk, *Belasting*, pp. 305–6.

93
Arickx, ‘De armendis’, p. 177.

94
Habets, ed., ‘Chronijk’, p. 43.

95 Other frequent terms include ‘*pestilentiaele siecte*’ (pestilential sickness), ‘*heete siecte*’ (hot sickness), and ‘*gave gods*’ (the gift from God).

96 The exact places and sources in the dataset can be found in online app. S1.

97
Noordegraaf and Valk, *De Gave Gods*, p. 49.

98
Benedictow, *Black Death, 1346–1353*, p. 298.

99
Curtis, ‘Was plague an exclusively urban phenomenon?’.

100 On this debate, see Bolton, ‘Looking for *Yersinia pestis*’; Green, ‘Editor's introduction’.

101
Cohn and Alfani, ‘Households’; Whittles and Didelot, ‘Epidemiological analysis’; Alfani and Murphy, ‘Plague’; Curtis and Roosen, ‘Sex‐selective impact’.

102
Hufthammer and Walløe, ‘Rats’; Cohn, ‘Epidemiology’; Walløe, ‘Medieval and modern bubonic plague’, p. 71; Ayyadurai, Sebbane, Raoult, and Drancourt, ‘Body lice’; Welford and Bossak, ‘Body lice’. This issue is unresolved.

103 The literature now asserts a broader range of potential mammalian and flea vectors; Carmichael, ‘Plague persistence’, pp. 159–60.

104 This is in line with a narrative suggested in Campbell, *Great Transition*.

105
Nicholas, ‘Economic reorientation’.

106
Vermeersch, ‘De Zwarte Dood’, p. 17–18. [Correction added on 28 May 2018, after first online publication: Reference corrected to Vermeersch, ‘De Zwarte Dood’, p. 17–18.]

107 Ibid., pp. 18–19. [Correction added on 28 May 2018, after first online publication: Reference corrected to Ibid., pp. 18–19.]

108 Calculated from Bocquet, *Recherches*, p. 37.

109 The count of 1286 from Devillers, ed., *Cartulaire*; the count of 1365 from ADN, B, no. 1586.

110
Derville, ‘La population’, p. 524.

111 Both quotations from Naphy and Spicer, *Plague*, pp. 34–5. For a similar view, see Scott and Duncan, *Biology of plagues*, pp. 102, 113.

112 Hainaut cities in the database were Ath, Soignies, Maubeuge, Bavay, Chimay, Avesnes, Landrecies, Busigny, Caudry, Bouchain, Le Quesnoy, Condé, St Ghislain, Chièvres, Mons, Leuze, Lessines, Le Roeulx, Braine‐le‐Comte, Enghien, Halle, and Flobecq. Valenciennes did not appear in the mortmain. Cities were distinguished from the countryside by only including so‐called ‘*bonnes villes*’ (the richest localities, usually fortified) not subject to the fiscal hearth count. Hearth counts were only conducted in the countryside, and thus any locality in the mortmain that was also in the hearth count was rural; Arnould, *Les dénombrements de foyers*, pp. 67–8.

113 The urban–rural ratio only surpassed 1:1 three times (in 1360, 1381, and 1425), partially due to greater urban exclusion from mortmain assessment, but also as more people in late medieval Hainaut were still resident in the countryside.

114 Each locality's mortmain was compared to the average mortmain rate for the whole period 1349–1450, rather than against the ‘previous years’ rate calculated for early modern burials in, for example, Alfani, ‘Plague’; Curtis, ‘Was plague an exclusively urban phenomenon?’; Alfani and Ó Gráda, eds., *Famine*. This was due to the lack of data for calculating a ‘previous years’ rate for the Black Death, and too many gaps in the series for individual (especially rural) localities considered in isolation—where some localities are excluded entirely on the basis of having too few annual mortmain deaths. By using this method, the estimates for territorial spread may be pushed downwards, since general population movements play a greater role in calculating the 1349–1450 average than when calculating the ‘previous years’ average.

115
Derville, ‘La population’, pp. 525–7.

116
Vangassen, ‘De pestepidemieën’, p. 8.

117 ARB, De Nelis, nos. 51–60.

118
Derville, ‘La population’, p. 526.

119 ‘Territorially pervasive’ is a term first introduced in Alfani, ‘Plague’, p. 408.

120
Alfani, ‘Plague’; Voigtländer and Voth, ‘Three horsemen of riches’.

121 For example, the figures for Leiden given in van Bavel and van Zanden, ‘Jump‐start’, pp. 507, 529–30. For Flanders more generally, see van Bavel, *Manors and markets*, p. 280. For Bruges, see Murray, *Bruges*, pp. 86, 106. For fast population recovery, increased urbanization, and weak Black Death logic, see Pamuk, ‘Black Death’, pp. 305, 308; Christakos et al., *Interdisciplinary public health reasoning*, pp. 222–3, 242–3.

122 On this migration process as a component of post‐plague urban resilience, see Stabel, ‘Working alone?’, p. 40; Dumolyn, ‘Population’, p. 63.

123
Vermeersch, ‘De Zwarte Dood’, p. 18. [Correction added on 28 May 2018, after first online publication: Reference corrected to Vermeersch, ‘De Zwarte Dood’, p. 18.]

124 Ibid., p. 102. [Correction added on 28 May 2018, after first online publication: Reference corrected to Ibid., p. 102.]

125
Blockmans et al., ‘Tussen crisis en welvaart’, p. 52.

126
Desportes, ‘Réceptions’, p. 556.

127
Thoen, ‘Commercial survival economy’, p. 120.

128
Cohn, ‘After the Black Death’.

129
van Bavel, *Manors and markets*, pp. 211–12.

130 See the comparisons made in van Bavel, ‘Markets’, pp. 504–5; Curtis, ‘Florence’.

131
Benedictow, *Black Death, 1346–1353*; Slack, *Plague*.

132
van Bavel and van Zanden, ‘Jump‐start’; van Bavel, *Manors and markets*.

133 de Moor and van Zanden, ‘Girl power’, pp. 16–17; Carmichael, de Pleijt, de Moor, and van Zanden, ‘European marriage pattern’, p. 201.
