# Supplementary material for: The ‘light touch’ of the Black Death in the Southern Netherlands: an urban trick?
Source: Econ Hist Rev. 2018 Feb 5;72(1):32–56. doi: 10.1111/ehr.12667 (PMC6472643; doi:10.1111/ehr.12667)
Supplement: Supplementary file 1 — S1. Plague mentions in the Low Countries sources, 1349–1499 [file EHR-72-32-s001.docx]

**Supporting Information 1 (S1). Plague mentions in the Low Countries sources, 1349-1499^[[1]](#footnote-0)^**

**KEY**

**Regions:**

Br = Duchy of Brabant; Ha = County of Hainaut; PoC = Pale of Calais; Gu = Duchy of Guelders; Fr = Frisia; Lo = County of Loon; Fl = County of Flanders; Ov = Oversticht; Ar = County of Artois; Lu = Duchy of Luxembourg; Ca = Cambrésis; To = Tournaisis; Ho = County of Holland; Ne = Nedersticht; Na = County of Namur; Li = Prince-Bishopric of Liege; LvA = Land van Aalst; Lim = Duchy of Limburg; UG = Upper Guelders; Bo = Boulonnais; Ze = County of Zeeland; Me = Lordship of Mechelen; Be = County of Bentheim; Cl = Duchy of Cleves; Jü = Duchy of Jülich; LvW = Land van Waas; VA = Vier Ambachte

**Archives**

ARB – Algemeen Rijksarchief Brussel (Brussels)

ACM - Archives Communales Mons

ADN - Archives Départmentales du Nord (Lille)

ADPdC - Archives Départementales Pas-de-Calais (Arras)

ARA - Archives Municipales d’Arras

AML - Archives Municipales de Lille

AMSO - Archives Municipales Saint-Omer

BASO - Bibliotheque d’Agglomeration de Saint-Omer

BHIC - Brabants Historische Informatie Centrum (Den Bosch)

ELO – Erfgoed Leiden en Omstreken

HCOvD – Historisch Centrum Overijssel vestiging Deventer

HUA - Het Utrechts Archief (Utrecht)

RA – Rijksarchief te Antwerpen (Antwerp)

GA – Gelders Archief (Arnhem)

GV – Gemeentearchief Venlo

RAZ – Regionaal Archief Zutphen

RB - Rijksarchief te Brugge (Bruges)

RK - Rijksarchief te Kortrijk (Courtrai)

SG – Stadsarchief Gent (Ghent)

SB - Stadsbibliotheek Brugge (Bruges)

SK – Stadsarchief Kampen

WBA – West Brabants Archief (Bergen-op-Zoom)

**1349-52**

| **Region** | **Quantifiable evidence of raised mortality** | **Descriptive mentions of plague in the contemporary sources** | **Descriptive mentions of plague in later sources** |
| --- | --- | --- | --- |
| Ha; Br; PoC; Gu; Fr; Lo; Fl; Ov; Ar; Lu; Ca; To Ho; Ne; Lim; Li; Na; | Lille (1); Ghent (2); Izel (3); Esquerchin (4); Aduard (5); Bruges (6); Ypres (7); Ath (8); Mons (9); Maubeuge (10); Soignies (11); Chièvres (12); Cambrai (13); Bapaume (30); St.-Omer (31); Aire (32); Kennemerland (33); Utrecht (34); Deventer (25); Béthune (26); Douai (44) | Sint-Truiden (14); St. Bertin (15); Wittewierum (16); Bavay (35); [Limburg] (39); Liege (40); St.-Omer (42); Béthune (29); Lille (38); Hesdin (37); Arras (24); Bas-Warneton (23); Hannut (22); Nieuwport (43); Zwolle (46) | [Frisia] (17); Valenciennes (18); Tournai (19); Arras (20); Ghent (36); Antwerp (41) ; Namur (27); Arnhem (28); Gorinchem (21); Amay (45) |

1. M. Aubry, ‘Les mortalités lilloises, 1328-69’, *Revue du Nord*, 65 (1983), p. 333. The mortality of *rentiers* between 1328 and 1369 shows the third highest spike in the period. A. Derville, ‘La population du Nord au Moyen Age’, *Revue du Nord*, 80 (1998), p. 525. For the hospital of St. Saveur, two documents from 1349 and 1355 show out of 65 living tenants, 45 had disappeared (69%), and for the countryside 12 out of 34 had disappeared (32%). Actual source: ADN, Archives Hospitalières de Lille, VI E7, fo. 9v.
2. J. Vermeersch, ‘De Zwarte Dood in Vlaanderen en Europa, economische impact en politieke reacties’ (unpub. MA thesis, Univ. Ghent, 2015), p. 15 ; 7 out of 36 names struck through indicating death, when in previous years this was usually one (1349).
3. A. Bocquet, *Recherches sur la population rurale de l’Artois et du Boulonnais pendant la période bourguignonne, 1384-1477* (Arras, 1969), p. 37. Households decline 26% between 1347 and 1351.
4. Ibid., 37. Households decline 18% between 1347 and 1351.
5. J. Mol, ed., *De abtenkroniek van Aduard: studies, editie en vertaling* (Hilversum, 2010), p. 30. The abbey chronicles suggest 92 deaths of ‘*scholares*’, and 46 out of roughly 100 monks in 1350 (46% death rate).
6. J. Vandeburie, ‘De Zwarte Dood te Brugge. Een status questionis en enkele nieuwe beschouwingen‘, *Handelingen van het Genootschap voor Geschiedenis te Brugge*, 147 (2010), pp. 269-308. Calculated total mortality rate of 35%.
7. Vandeburie, ‘De Zwarte Dood’.
8. W. Blockmans, ‘The social and economic effects of plague in the Low Countries, 1349-1500’, *Revue Belge de Philologie et d’Histoire*, 58 (1980), p. 838.
9. G. Sivéry, ‘Le Hainaut et la Peste Noire’, *Mémoires et Publications de la Société des Sciences, des Arts et des Lettres du Hainaut*, 79 (1965), p. 438.
10. Over 100 mortmain dead in 1349 compared to average between 1349 and 1450 of roughly 33. ADN, B 12122.

Sivéry, ‘Le Hainaut’, p. 437.

A total of 64 mortmain dead in 1349 compared to average between 1349 and 1450 of roughly 29. ADN, B 12122.

Derville, ‘La population’, p. 524. Excess mortality of nearly 15% for the chaplains of the cathedral and over 35% for their farmers. Actual source: ADN, 4G, nos 6849, 6853.

E. Lavigne, ed., *Kroniek van de abdij van Sint-Truiden, 1138-1558. Vertaling van de Gesta Abbatum Trudonesium*, II (Assen, 1988), p. 187.

B. Delmaire, ‘Contribution à l’étude de la peste au bas moyen âge. Un fragment de compte inédit de l’abbaye de Saint-Bertin pour Bas-Warneton’, *Mémoires de la Société d’Histoire de Comines-Warneton et de la Région*, 11 (1981), p. 48.

H. Jansen, ed., *Kroniek van het klooster Bloemhof te Wittewierum* (Hilversum, 1991).

Worp of Thabor (Prior), *Chronicon Frisiae libri tres*, ed. J.G. Ottema (Leeuwarden, 1847), p. 190. Sixteenth-century chronicles.

J-N. Biraben, *Les hommes et la peste en France et dans les pays Européens et Méditerranées*, I (Paris, 1975), p. 76.

C. Dury, ‘L’évolution démographique de Tournai au Moyen Age’, in *Autour de la ville en Hainaut. Mélanges d’archéologie et d’histoire urbaines offerts à J. Dugnoille et à R. Sansen* (Ath, 1986), pp. 185-203.

A. Guesnon, ed., *Inventaire chronologique des chartes de la ville d’Arras; documents* (Arras, 1863), no. 97. On 5 July 1352, Queen Jeanne gave financial aid to the city and mentioned the great mortality as reason for this: « *par la mortalité qui a esté par universel monde, ladite ville est si grandement amoindrie, tant de personnes et habitants comme de revenues et biens temporels, qu’elle est en voie de desolation ».*

C. van Zomeren, *Beschryvinge der stadt Gorinchem, en landen van Arkel...* (Gorinchem, 1755), p. 285; “*zo gevoelden deze Stad ook die zwaare straf...dat den Pastoor, Kapellaan, eenige Schepenen en wel vier hondert andere Menschen wierden weg gerukt*”.

G. Despy, ‘La ‘Grand Peste Noire de 1348’ a-t-elle touché le roman pays de Brabant?’, in *Centenaire de Séminaire d’Histoire Médiévale de l’Université Libre à Bruxelles, 1876-1976* (Brussels, 1977), p. 205. Two documents from 1360 referring to plague in Hannut.

Delmaire, ‘Contribution à l’étude de la peste’, fn. 13.

R. Berger, ed., *La nécrologie de la confrérie des jongleurs et des bourg d’Arras (1194-1361)* (Arras, 1963), no. 10. It was noted on 6 October 1349 that « *la mortalité était pour le temps moult grande en ladite ville* ».

Blockmans, ‘The social and economic effects of plague’, p. 843. The necrology of Lebuinus Church in Deventer had 52 deaths recorded with a highpoint of 25 in July, while in the previous 20 years there had been no more than 45.

Delmaire, ‘Contribution à l’étude de la peste’. Out of six aldermen, four died during the initial Black Death outbreak.

M. Galliot, *Histoire Générale, Ecclésiastique et Civile de la Ville et Province de Namur*, V (Namur, 1788-91), p. 4 « *Cette annee [1349] et la suivante, durant le regne du comte Guillame premier du nom, une cruelle peste ravagea toutes les provinces…Ce mal augmenta a Namur par le debordement de la Meuse et de la Sambre, par la raison que les eaux s’etant ecoulees, il resta sur le rivage, un limon corromptu et puant qui fortifia l’infection de l’air* ».

G. Leppink and R. Wientjes, *Het Sint Catharinae gasthuis te Arnhem in de eerste vier eeuwen van zijn bestaan (1246-1636)* (Hilversum, 1999).

O. Bled and D. Haigneré, eds., *Les chartes de Saint-Bertin d’après le Grand Cartulaire de Charles-Joseph Dewitte*, II (St. Omer, 1892), no. 1693.

Derville, ‘La population’, p. 524. Mortality rates suggested as high as 60%.

AMSO, Registre au Renouvellement de la Loi, no. 5. In 1349 33 ‘*changeurs*’ existed in the city; by 1352 only 13 remained, a loss of 61%.

Derville, ‘La population’, pp. 523-5. The number of ‘*echoites*’ of bastards and escheated property multiplied by 9, indicating roughly 33% mortality. Actual source: ADN, B, no. 13632.

D. de Boer, *Graaf en grafiek: sociale en economische ontwikkelingen in het middeleeuwse ‘Noordholland’ tussen 1345 en 1415* (Leiden, 1978), p. 34; spikes in deaths of serfs, bastards and foreigners in the accounts of the Count of Holland.

R. Rommes, ‘Op het spoor van de dood. De pest in en rond Utrecht’, *Jaarboek Oud-Utrecht* (1991), p. 118. Also mention of flagellants in 1351 in a sixteenth-century chronicle collection; J. Joosting, ed., ‘Cornelis Block’s kroniek van het regulierenklooster te Utrecht’, *Bijdragen en Mededeelingen van het Historisch Genootschap*, 16 (1895), p. 23.

ADN, B, no. 10817. An account from 4 August 1349 mentions the burning of Jews after plague.

A. van Heule, ed., *Memorieboek der Stad Ghent van ’t jaar 1301 tot 1793*, I (Ghent, 1852), p. 59; “*In dit jaere stont eene peste oppe, die wel dry jaren duerde, zoo dat wel dhelft van den volcke op de werelt zijnde starf*” (1345). Followed in 1349 (pp. 67-8) by a long description of a large flagellant movement in the city.

ADPdC, Arras, A85, nos 8, 10.

AML, AA142/2633, nos 1650, 1652.

T. Elhen von Wolfhagen, *Die Limburger Chronik*, ed. O. Hermann Brandt, IV (Jena, 1922), no. 31; “*In Limburg starben ohne die kinder mehr denn 2400 menschen. In dem jahre 1350 hörte das sterben auf*“ (1349).

F. Graus, *Pest – Geißler – Judenmorde* (Göttingen, 1987), p. 38. Recalls the songs translated into French from German in the town of Liege by the flagellants there. Liege is said to have escaped this outbreak of Black Death according to C. Renardy, ‘Un témoin de la Grande Peste: Maître Simon de Couvin, chanoine de Saint-Jean l’Evangéliste à Liège (f 1367)’, *Revue Belge de Philologie et d’Histoire*, 52 (1974), fn. 8; but ‘*pestis*’ is mentioned in E. Bacha, ed., *La chronique liégoise de 1402* (Brussels, 1900), pp. 341-2.

J.C. Diercxsens, *Antverpia Christo nascens*, II (Antwerp, 1774), p. 104.

A. Derville et al., *Historie de Saint-Omer* (Lille, 1981), p. 73. Communal graveyards were pressed to the limit and so on 16 July 1349, the city administration asked the vicars of the bishop to open a new cemetery in the parish of St. Michel. Actual source: AMSO, B 241.36.

In an agreement drawn up between the coastal town of Nieuwpoort and Furnes-Ambacht on 3 April 1350, it is explicitly mentioned that plague mortality was the cause of inhabitants moving from the villages of Leke and Klerken to the town; L. Gilliodts-Van Severen, ed., *Coutumes des pays et Comté de Flandre: Quartier de Furnes*, II (Brussels, 1872), p. 119.

Derville, ‘La population’, p. 524. A third of the city’s aldermen died, and six out of nine vicars of the college of Saint-Amé.

Bacha, ed., *La chronique liégeoise*, p. 343; ‘*mortalitas*’ mentioned in 1351.

F.C. Berkenvelder, ed., *Zwolse regesten*, I (Zwolle, 1980), no. 3; “*ten gevolge van de nu heersende epidemie bij het klooster begraven*” (12 January 1351).

**1358-62**

| **Region** | **Quantifiable evidence of raised mortality** | **Descriptive mentions of plague in the contemporary sources** | **Descriptive mentions of plague in later sources** |
| --- | --- | --- | --- |
| Ne; Fl; Ca; Ar; Ho; Fr; Gu; Na; Li; Ha | Lille (1); Cambrai (2); St. Omer (3); Beuvry (4); [Rijnland] (5); Ghent (6); Ypres (15); Bergen-op-Zoom (17) | Utrecht (city & countryside) (7); Rhenen (8) | Arras (9); [Frisia] (10); Namur (11); [Liege] (12); [Hainaut] (13); Ghent (14); Huy (16) |

1. Aubry, ‘Les mortalités lilloises’, p. 333. Spike in mortality of *rentiers* (annuity information). Actual source: ADN, CC, no. 16012 (1317-1318) - 16095 (1368-1369).
2. Derville, ‘La population’, pp. 525-6. Chaplains of the cathedral and farmers both reaching surplus of mortality of roughly 33%. Actual source: ADN, 4G, nos 6856-8. Also the accounts of the St.-Julien hospital of Cambrai mention plague in 1360: A. Derville, ‘L’hôpital Saint-Julien de Cambrai au XIVe siècle: étude économique’, *Revue du Nord*, 70 (1988), pp. 285-318.
3. Bled & Haigneré, eds., *Les chartes de Saint-Bertin*, no. 1710. Between 1357 and 1366 there was a decline of the number of communicants by roughly 48% (10,200 to 5,350). The abbot of the St.-Bertin abbey in St.-Omer reported in September 1361 that « *son [the plague] trespassament de jour en jour pour la tres grant pestilence de mortalité* ». Furthermore, in St.-Denis the number of *pascalisants* dropped from 2500 to 1200, in St.-Sépulcre from 2600 to 1300-1400, in St.-Aldegonde from 1800 to 1000, and in St.-Margueritte from 3300 to 1800; see A. Derville, ‘Le nombre d’habitants des villes de l’Artois et de la Walloon Flanders (1300-1450)’, *Revue du Nord*, 65 (1983), pp. 281-2. Actual sources: AMSO, B 46, passim; B 53, nos 5-10, 49; B 54, nos 4-5; B 55, nos 1, 5.
4. Derville, ‘La population’, p. 526. Actual source: ADN, B, nos 15022-42.
5. R. Ladan, *Gezondheidszorg in Leiden in de late Middeleeuwen* (Hilversum, 2012), p. 45.
6. SG, Archief H. Geesttafel van St. Niklaaskerk, no. 30. Clear increase in the number of purchased coffins compared to following years. Furthermore, there is a spike in the inventories after death in the years 1360/1, 106 and 130 acts respectively compared to 37 on average in the previous decade; L. Wynant, ed., *Regesten van de Gentse staten van goed: 1349-1400*, I (Brussels, 1979), pp. XVIII-XXI.
7. C. Rutgers, *Jan van Arkel, bisschop van Utrecht* (Groningen, 1970). Actual plague year mentioned was 1359.
8. Ibid. Actual plague year mentioned was 1359.
9. ADPdC, A92, no. 5.
10. P. Winsemius, *Chronique ofte Historische geschiedenisse van Vrieslant* (Franeker, 1622), p. 205.
11. Galliot, *Histoire Générale*, V, p. 4; « *Une maladie pestilentielle qui s’étoit manifestée par plusiers reprises dans le pays de Liège et dans la Hesbaye, vint infecter le comté de Namur, où elle fit un terrible ravage, parmi les hommes et les bestiaux. On croit que l’hiver, qui fut très rude cette année, ne contribua pas peu à purifier l’air, et à dissiper le souffle contagieux* ».
12. Ibid. Also « *très grande mortalité de la bouche* » (1362) in S. Balau & E. Fairon, eds., *Chroniques liégeoises*, II (Brussels, 1931), p. 190.
13. Ibid. Also large rise in mortalities in the mortmain; ADN, B 12130-2.
14. Van Heule, ed., *Memorieboek*, I, p. 84; “*Item, in dit jaer was't eene groote sterfte van der haestichede; de lieden waren snaevonts fraey ende snuchtensdoot*” (1360).
15. Vandeburie, ‘De Zwarte Dood’. Based on issue payments.
16. Bacha, ed., *La chronique liégeoise*, p. 347; “*maxima mortalitas…ex epydimia qui tune regnabat in mundo*” (1360).
17. M. Green, <<https://contagions.wordpress.com/2016/06/29/plague-dialogues-monica-green-and-boris-schmid-on-plague-phylogeny-ii/>> (2016). Bio-archaeological evidence of *Yersinia pestis* in burial site attributable to these years. Originally erroneously dated to the Black Death period of 1349/50; S. Haensch et al., ‘Distinct clones of *Yersinia pestis* caused the Black Death’, *PLoS Pathogens*, 6 (2010), e1001134.

**1367-9**

| **Region** | **Quantifiable evidence of raised mortality** | **Descriptive mentions of plague in the contemporary sources** | **Descriptive mentions of plague in later sources** |
| --- | --- | --- | --- |
| Fl; Ar; Ca; Ov; Ho; To; LvA; Br; Li; Ne; Ze; LvW; Na; Ha | Lille (1); Beuvry (2); Béthune (3); Cambrai (4); Ghent (5); Leiden (8); Tournai (9); Oudenaarde (10); [Land van Heusden] (11) Vlijmen (14); Hazerswoude (15); Zegwaart (17); Zoetermeer (18); Zoeterwoude (19); Gelderswoude (20); Schoonhoven (21); Beveren (22); Ypres (24); Petegem (25); Brekel (28); [Hainaut] (31) | Lillers (12); Namur (26); Lille (27); | Liege (13); [Ghent-Flanders-Artois-Brabant-Holland-Zeeland] (23); Tournai (29); Gorinchem (30); [Brabant] (16); Leiden (6); Utrecht (7); |

1. Aubry, ‘Les mortalités lilloises’, p. 333. Largest spike in mortality of *rentiers* (annuity information) between 1329 and 1369. Actual source: ADN, CC, nos 16012 (1317-1318) - 16095 (1368-1369). Also ADN, Archives Hospitalières de Lille, VI E9; information from St. Saveur hospital.
2. Derville, ‘La population’, p. 526. Normal rate of ‘reliefs’ was 8.25 per year. In 1368 this shot up to 163 (minimum). Actual source: ADN, B, nos 14601-16. According to Derville’s calculations, in 166 days more than 40% of the adult population in Beuvry had died. Idem., p. 526.
3. Ibid., p. 526. Normal rate of ‘reliefs’ was 8.62 per year. In 1367 this shot up to 52 and in 1368 this was 126. Actual source: ADN, B, nos 14601-16.
4. Ibid., p. 527. There was a recorded death rate of 10% for the cathedral chaplains and 25% for their farmers. Actual source: ADN, 4G, nos 6859-63. Also mentioned in the accounts of St.-Julien hospital of Cambrai in 1369-70; Derville, ‘L’hôpital Saint-Julien’.
5. SG, Archief H. Geesttafel van St. Niklaaskerk, no. 30. Clear increase in the number of purchased coffins compared to following years. Furthermore, there is a spike in the inventories after death in 1368-70, 160, 193 and 171 acts respectively compared to 75 on average in the previous decade; Wynant, ed., *Regesten*, I, pp. XVIII-XXI.
6. ELO, Archieven van het Sint Pancraskerk, Antiquum registrum A, no. 415, fo. 29; “*In de loop van het jaar 1369 nam de genoemde pestilentie of epidemie ergst toe in de stad Leiden en omliggende plaatsen, zozeer dat in dat jaar meer dan drieduizen mensen ... uit genoemde stad aan genoemde ziekte op ellendig wijzen zijn overleden; onder welke mensen van beiderlei kunnen en uiteenlopende leeftijd de meesten toch kinderen en jonglieden waren; en bijna alle zwangere vrouwen stierven...*”. Also H.G. Hamaker, ed., ‘Historische aanteekeningen in het ‘Memoriale fautorum capitulli Sti Pancratii’ te Leiden, 1367-1408’, *Bijdragen voor Vaderlandsche Geschiedenis en Oudheidkunde*, 6 (1869/70), p. 127 (1368); *“...gravis pestilentia seu morbus quem physici epidemiam vocant...*”; again in 1369; Idem, p. 128; *“...dicta pestilentia seu epidemia gravissime...*”.
7. *Kronijk van Arent toe Bocop* (Utrecht, 1860), p. 355 (1368). Also in N. Bruch, ed., *Chronographia Johannis de Beke* (The Hague, 1973), p. 336; *“…anno Domini mccclxviii magna mortalitas seu epidimia fuit in civitate Traiectensi…*’ (1368).
8. Ladan, *Gezondheidszorg*, p. 45; De Boer, *Graaf en grafiek*, pp. 74-5. Peak in the number of dead ‘*fautores*’ of the St. Pancras church in Leiden in 1369 (50 dead), when only 8 in 1368 and 5 in 1367. In later years only 5 in 1370, 3 in 1371 and 8 in 1372. Mortality in 1369 shows seasonal pattern associated with plague – 0-2-0-2-5-3-12-17-3-5-1-0 (per month).
9. Blockmans, ‘The social and economic effects of plague’, p. 851.
10. E. Thoen, *Landbouwekonomie en bevolking in Vlaanderen gedurende de late Middeleewen en het begin van de Moderne Tijden. Testregio: de kasselrijen van Oudenaarde en Aalst* (Ghent, 1988), pp. 1141-53.
11. P. Hoppenbrouwers, *Een middeleeuwse samenleving. Het Land van Heusden (ca. 1360-ca. 1515)* (Wageningen, 1992), p. 58.
12. ADPdC, A880. « *Le mortuoire dura del entrée d’auost jusqu’à le Candeler* ».
13. G. Guttmann, ed., ‘Jean à la Barbe. Traité sur la peste’, in *Die Pestschrift des Jean à la Barbe (1370)* (Berlin, 1903).
14. Hoppenbrouwers, *Een middeleeuwse samenleving*, p. 60.
15. De Boer, *Graaf en grafiek*, pp. 98-100, 347; decline of 43.3% in names between the inquisition of 1369 and a ‘naamlijst’ from 1371.
16. Despy, ‘Grand peste noire’, p. 205. Fn. 27 makes reference to the research of a one Uyttebrouck who noted plague in « *Icomptes de la recette générale de Brabant* ».
17. De Boer, *Graaf en grafiek*, pp. 101-2; decline of 26.8% in names between the inquisition of 1369 and a ‘naamlijst’ from 1371.
18. Ibid., pp. 101-2; decline of 30% in names between the inquisition of 1369 and a ‘naamlijst’ from 1371.
19. Ibid., pp. 102-5; decline of 29.2% in names between the inquisition of 1369 and a ‘naamlijst’ from 1371.
20. Ibid., pp. 102-5; decline of 46.5% in names between the inquisition of 1369 and a ‘naamlijst’ from 1371.
21. C.J. de Lange van Wijngaarden, *Geschiedenis der heeren en beschrijving der stad van der Goude*, I (The Hague, 1813), p.754; “*rekende men te Sinte Gheerdenberghe, om der starften wille die tScoenhoven groot was*” (22 November 1369).
22. W. Vangassen, ‘De pestepidemieën na 1350, voornamelijk deze van 1400 en 1438 in Vlaanderen en Henegouwen’ (unpub. Ph.D. thesis, Univ. Ghent, 1952), p. 8. Increased death of servants according to accounts of 1368/9. Actual source: ARB. De Nelis, nos 8-15, Rekeningen Lopen van St. Jansmesse tot St. Jansmesse.
23. Van Heule, ed., *Memorieboek*, I, p. 91; “*Item, in dit jaer was't groote sterfte in Vlaenderen, Brabant, Hollant, Zeelant en in Artoeys*” (1367). Also for Flanders, Brabant and Picardy; Jan van Dixmude, Dits de Cronike, *Corpus croniconun Flandriae*, ed. J. J. De Smet (Brussels, 1856), p. 232, 618; “*Anno MCCCLXVII fuit magna mortalitas in Flandria, Brabantia et Picardia, homines subitanea ac morte improvien moribantur*”.
24. Vandeburie, ‘De Zwarte Dood’. Based on issue payments.
25. Vangassen, ‘De pestepidemieën’, pp. 8-9. Clear peak in the ‘*Ontfaen van Doetcoepe*’ between June 1368 and June 1369 (13 compared to average of 4), compared to previous and following years. Actual source: ARB, De Nelis, nos 51-60.
26. L. Génicot, *L’économie rurale Namuroise au bas Moyen Age (1199-1429). 1. La seigneurie foncière* (Namur, 1943), p. 285. In the accounts of the hospital of Schayn it was noted that « *encore doit on rabatre les parties d’espialt qui s’ensuivent dont les hirretayes sunt démenées et sunt à present vaghe par le mortalieit de gens et de chevaux* ».
27. ADN, 16G (Chapitre Saint-Pierre de Lille), no. 1034; proceedings of the chapter against Jean de Ferlin with mention of the plague outbreak of 1369.
28. De Boer, *Graaf en grafiek*, pp. 108-11.
29. Van Dixmude, *Corpus croniconun Flandriae*, III, p. 240; « *A le paque l’an 1367 commencha à Tournay I grant mortolle, qui dura dusques au Noël ensievant et fu le mortelle si grant que a miervelle et fist on plusieurs ordonnanches sur le fait dudit mortolle*».
30. A. Kemp, *Leven door doorluchtige heeren van Arkel ende jaarbeschrijving der stad Gorinchem* (Gorinchem, 1656), p. 89, 102.
31. High mortalities in the mortmain; ADN, B 12138.

**1380-2**

| **Region** | **Quantifiable evidence of raised mortality** | **Descriptive mentions of plague in the contemporary sources** | **Descriptive mentions of plague in later sources** |
| --- | --- | --- | --- |
| Fl; Ca; Ho; LvA; Br; Gu; Ne; Na; Ov; Lu; Ar; Ha; Li | Douai (1); Cambrai (2); [Kennemerland] (3); [Rijnland] (4); Haarlem (5); Leiden (7); Oudenaarde (8); Breda (9); Izel (16); Esquerchin (17); Lille (18); Ghent (20); [Hainaut] (22); | [Guelders] (10); Leiden (15); Lille (19); Deventer (11) | Ghent (14) Deventer (13); [Flanders] (21); [Brabant] (12); Den Haag (6); Bilzen (23) |

1. J. Deregnaucourt, ‘Autour de la mort à Douai: attitudes pratiques et croyances, 1200-1500’ (unpub. Ph.D. thesis, L’Université Charles de Gaulle, 1992), I, p. 60. Clear spike in the number of wills written.
2. H. Neveux, ‘La mortalité des pauvres à Cambrai (1377-1473)’, *Annales de Démographie Historique* (1968), p. 79.
3. De Boer, *Graaf en grafiek*, p. 64; raised numbers of ‘*besterften*’ in 1382 (23) and 1383 (45) when the previous decade offered no more than 9.
4. Ibid., p. 64; as above.
5. Ibid., pp. 83-4 (in 1381).
6. Ibid., p. 67; noted on 18 October 1382 that “*omt ghestant vander sterfte aldaer te vernemen ende mire vrouwen aen te brengen*”.
7. Ladan, *Gezondheidszorg*, p. 45.
8. Thoen, *Landbouwekonomie*, pp. 1141-53.
9. F. Gooskens, ‘Pestepidemieën in Breda tijdens de middeleeuwen (1382-1535)’, *Jaarboek De Oranjeboom*, 39 (1986), pp. 18-54.
10. J. van Veen, ‘De pest en hare bestrijding in Gelderland, in het bijzonder te Arnhem’, *Bijdragen en Mededelingen Gelre*, 6 (1903), pp. 1-66.
11. Death of Geert Grote on 20 August 1384 through plague mentioned in the *cartusia maior* chronicles of 1385; H. Scholtens, ‘De priors van het kartuizerklooster Monnikhuizen bij Arnhem’, *Archief voor de Geschiedenis van het Aartsbisdom Utrecht*, 56 (1932), p. 11. Also D.A. Brinkerink, ed., ‘Dit is genamen uyten leven onses weerdigen vaders meyster Gerijt die Grote’, <<http://www.dbnl.org/tekst/_lev018biog01_01/_lev018biog01_01_0002.php>>, p. 423; *“...waert hij begaeft van Gode mitter pestelencie*” (1384).
12. Despy, ‘Grand peste noire’, p. 205; fn. 27 makes reference to the research of a one Uyttebrouck who noted plague outbreaks of 1382-3 in « *Icomptes de la recette générale de Brabant* ».
13. P. Molhuijsen, ‘Vroegere ongezondheid van Deventer’*Overiijsselsche Almanak voor Oudheid en Letteren*, 7 (1842), p. 64. In 1384 according to the 18th-century ‘*Kronijk van Deventer*’ by Sef Moonen.
14. Van Heule, ed., *Memorieboek*, I, p. 112; “*Item, in dit jaer was 't groote aertbevinghe ende sterfte, ende midts der orloghe stont de stede Ghent langhe zonder heere*” (1381).
15. B. Leverland, *St. Pancras op het Hogeland: kerk en kapittel in Leiden tot aan de Reformatie* (Hilversum, 2000), p. 99. Priest Pieter Jacobsz fled to Solesmes in Hainaut (where he died in 1380) on account of the raging plague in Leiden. Also ELO, Archief van de Sint Pancraskerk, Antiquum Registrum A, no. 415, fo. 76; notes over the plague in 1381.
16. Bocquet, *Recherches sur la population rurale*, p. 37, 53, 80. Households decline almost 26% between 1377 and 1385.
17. Ibid., p. 37, 53, 80. Households decline 45% between 1377 and 1385.
18. In Lille’s St.-Saveur hospital, there were no recorded deaths of poor people between 1371 and 1375, while a large number were found in the deceased of 1381; ADN, Archives Hospitalières de Lille, VI E, no. 10.
19. The city of Lille was said to be depopulated in 1382, on account of a previous mortality; ADN, Inventaire Godefroy, 11, no. 1100, fo. 104.
20. There is a spike in the inventories after death in 1383, 152 acts compared to 75 on average in the previous decade; Wynant, ed., *Regesten*, I, pp. XVIII-XXI. Issue rights also increase, but it is difficult to compare with surrounding years which are also crisis periods; Vangassen, ‘De pestepidemieën’, p. 26.
21. A. De Roovere, *Dits die excellente cronike van Vlaenderen…* (Ghent, 1531), fo. 70.
22. Large rise in mortalities in the mortmain; ADN, B 12147; ARB, I 004 17869.
23. Bacha, ed., *La chronique liégeoise*, p. 394; “*magna mortalitas hominum*” in 1382.

**1400-2**

| **Region** | **Quantifiable evidence of raised mortality** | **Descriptive mentions of plague in the contemporary sources** | **Descriptive mentions of plague in later sources** |
| --- | --- | --- | --- |
| Ar; Ha; To; Fl; Ca: Ov; Ho; Gu; Br; Ne; LvA; Na; Fr; Ze; Lim; Li; Cl | Arras (1); Mons (7); Tournai (8); Douai (9); Lille (10); Cambrai (11); Ghent (12); Leiden (15); Breda (20); Oudenaarde (21); [Amstelland] (24); [Waterland] (25); [Kennemerland] (26); [Delfland] (27); [Schieland] (28); Gouda (29); Schoonhoven (30); Woerden (31); Aalst-Geraardsbergen (40); Bruges-Franc of Bruges (41); Courtrai (42); Sluis (43); [Hainaut] (44); Liege (46); Jemeppe (47) | Fampoux (3); [Bailliages of Arras, Avesnes, Aubigny and Quiéry] (4); [Artois] (5); Mons (6); Deventer (14); [Veluwe] (19); Zwolle (38); Tournai (35) ; Harelbeke (34); Middelburg (17); Dunkirk (13); St. Omer (39); Vivegnis (48); [Nederbetuwe] (50); Almelo (52); Frenswegen (53) | Arras (2); Amersfoort (16); [Frisia] (23); Ghent (32); Bruges (33); Deventer (37); Binche (36); Namur (22); [Rijnland] (18); Maastricht (45); Kampen (49); Bommel (51); Geldern (54) |

1. ADPdC, Archives Hospitalières d’Arras, no. 1, E10. From 1 October 1399 to 30 September 1400, the hospital Saint-Jean of Arras buried 246 bodies, while the normal number of burials was rarely more than 50 per year. Unfortunately this account is not part of a series, and the previous preserved dates are 1371-1373 and the following 1414-1415.
2. Guesnon, ed., *Inventaire chronologique des chartes de la ville d’Arras*, no. 151. « *Le peuple de nostre dicte ville est moult diminué par la pestilence et mortalité qui, par l’espace de trois ans ou environ, a esté continuellement en ycelle nostre ville ».*
3. Bocquet, *Recherches sur la population rurale*, p. 72. On 8 March 1402, Duchess Marguerite ordered her receiver in Fampoux to repay the inhabitants of the village a tax they had paid earlier because of their poverty and the recent mortality crisis; « *Et pour la mortalité icelle nostre ville soit moult despeuplée et amenrie de manans et residens et leurs tenemens tournez en ruine ».* Actual source*:* ADN, B, no. 1872.
4. Ibid., p. 72. The duke lowered the aide/taxation in the Bailiwicks of Arras, Avesnes, Aubigny and Quiéry; « *ou nos subgés sont trés fort diminuez par la mortalité* *»*. Actual source*:* ADN, B, no. 1874.
5. Ibid., p. 73. The duchess had a personal encounter with plague and we learn that she hastened to Rémy; « *pour logier, par certain temps, sa personne et son etat et ses gens, ouquel temps la mortalité régnoit en nostre ville d’Arras, en laquelle elle faisoit sa residence lors* ». Because she still did not feel safe, she left for Douai « *pour cause de la mortalité qui estoit au comté d’Artois…*». Actual source*:* ADN, B, no. 1901.
6. G. Decamps, ‘La maladie contagieuse de 1400-1401 à Mons et dans le Hainaut’, *Annales du Cercle Archéologique de Mons*, 41 (1912), p. 139. « *Vers le mois de juiny s’élèva en la ville ung air pestilentieux et causa grand morteile et mourut bien ung tierch des boines gens y demorans tant du petit que du grant et des mieux moyennés. Le capitle perdit en cest an plusieurs canoines capellains et maisnies* ». Also Idem., p. 145; on 6 January 1401 the aldermen assemble and go to the Count of Ostrevant to « *s’excusant de ne pas ester venus plus tost à cause de la piteux mortalitet qui avoit estet en la ville* ».
7. Ibid. p. 139. The disease had a severe impact as seen from the expenses in the account from ‘*la grande aumone’* that ran from St. Jan Baptist 1400 until St. Jan Baptist 1401 and paid for 438 coffins. For a normal year this was only 50 on average. Better off citizens also seemed to have been afflicted as the ‘*Comptes des draps de mort ou de bouguerants*’ from St. Waudru lists 177 dead, although the author does not give a comparative figure for ‘normal’ years; Idem., p. 139.
8. H. Vandenbroeck, *Extraits analytiques des anciens registres des Consaux de la ville de Tournai (1385-1422)*, I (Tournai, 1861), p. 47. For the year 1400, there were 339 wills in the Tournai Archive, while for a ‘normal’ year this was never more than 80 (a rise of 424%).
9. The number of testaments reached a peak in 1400; Deregnaucourt, ‘Autour de la mort à Douai’, I, p. 60.
10. In the hospital of St.-Saveur in Lille, two nuns died as well as 40 patients. ADN, Archives Hospitalières de Lille, VI E 11.3.
11. Derville, ‘La population’, p. 67. Farmers’ excess mortality was roughly 24%. Actual source: ADN, 4G, nos 68884-7.
12. Vangassen, ‘De pestepidemieën’, p. 26. Based on the issue rights. Furthermore, there is a spike in inventories after death in 1400-1 of 124 and 169 compared to 68 on average in the previous decade; Wynant, ed., *Regesten*, I, pp. XVIII-XXI.
13. S. Curveiller, *Dunkerque: ville et port de Flandre à la fin du Moyen Age: à travers les comptes de bailliage de 1358 à 1407* (Lille, 1989), p. 19. (In 1397-8). Original source found in ADN, B, no. 5984.
14. T. Mertens, ‘Rondom het sterfbed van Lubbert ten Busch. De Moderne Devoten en de pest te Deventer in 1398’, in *De Pest in de Nederlanden: medisch-historische beschouwingen 650 jaar na de Zwarte Dood* (Brussels, 1999), pp. 141-58. (In 1398). Also Brinkerink, ed., ‘Dit is genamen uyten leven onses weerdigen vaders meyster’, p. 331, 338 (1398); “*waert begaeft mitter pestilencie*”.
15. Ladan, *Gezondheidszorg*, p. 233. In 1398 the memory book already mentioned deaths by *pest*. The number of benefactors increased to 21 in 1399 and 18 in 1400 – more than 50% over the norm. The number of graves given out in the St. Peters Church went from 10 in 1398/9 to 20 in 1399/1400 and to 30 in 1400/1, and then afterwards fell back to roughly 4 and 5.
16. C. van Kalveen, ‘Problemen rond de oudste geschiedenis van het fraterhuis en van het Nieuwe Gasthuis te Amersfoort’, *Jaarboek Oud-Utrecht* (1981), pp. 101-24.
17. F. van den Driest, ‘Hondenbaan: hondenslagers op Walcheren’, *Heemkundige Kring Walcheren*, 38.1 (2009), p. 38; in the years 1399/1400 were there an exceptional 252 dogs slaughtered (often response to plague).
18. De Boer, *Graaf en grafiek*, p. 67.
19. J. Kuys, ed., *De Tielse Kroniek. Een geschiedenis van de Lage Landen van de Volksverhuizingen tot het midden van de vijftiende eeuw, met een vervolg over de jaren 1552-1566* (Hilversum, 1983), pp. 137-8; the period 1400/1 described as “*die grote starft*”.
20. Gooskens, ‘Pestepidemieën’, p. 31. Necrology data.
21. Thoen, *Landbouwekonomie*, pp. 1141-53.
22. J. Borgnet, *Histoire du Comté de Namur* (Namur, 1850), p. 155. Also Galliot, *Histoire Générale*, V, p. 11; « *Sous le règne du comte Guillaume second du nom, la peste se fit de nouveau sentir à Namur, & y fit bien du dégar. La mortalité fut si grande, qu’on dut demolir l’école de le collégiale Notre-Dame, pour agrandir le cimetière de cette paroisse, qui ne pouvoit plus suffir pour y enterrer les morts* ».
23. P. Jacobson of Thabor, *Historie van Friesland* (Leeuwarden, 1973), pp. 9-11.
24. De Boer, *Graaf en grafiek*, p. 67
25. Ibid., p. 67.
26. Ibid., p. 67.
27. Ibid., p. 67.
28. Ibid., p. 67.
29. Ibid., p. 67.
30. Ibid., p. 67.
31. Ibid., p. 67.
32. Van Heule, ed., *Memorieboek*, I, p. 134; “*In dit jaer waren in Italien vergadert wel tachentich duusent mannen, dewelcke men hiet de Groote Compaingnie, ende onder desen hoop waren veele hertoghen, graven en baenderheeren, ende zy waeren ghecleet met witte cleederen en zy straften zeere de zonen der meynschen, ende ghemerct dat de sterfte in deze landen zeere regnierde zoo belofden zy elcken die onder huerlieder compaignie quamen dat zy vry zouden wesen van der siecte van der pestilentie*” (1399). And in 1401, (p. 136), *“[In 't zelve jaer was te Ghendt een groot pardoen het jaer van gratie ende 't was t' Onze Vrouwekercke te Ste. Pieters, en was doe een groote sterfte. (Pr. Ar.B.)] Item, in dit jaer was te Ghent groote sterfte ende al Nederlant duere*”.
33. SB, Kroniek van Vlaanderen, Handschrift 436, Band B, no. 38 (f°CLVIII r°b-v°a); “*Item int jaer van XIIIIC b, zo was te Roome groot aflaet ende was tjaer van gracien ende elkerlyc liep te Roome omme te hebbene aflaet a pena et a culpac. Ende int zelve jaer zachd men int weste staen // een planete ende een sterre met eenen langhen sterte. Ende int zelve jaer was al omme groot sterfte van der pestelencie*”.
34. Vangassen, ‘De pestepidemieën’, pp. 30-1. Between 1 October 1399 and 10 July 1400, the bailiff Francois Camphin dies, and then his successor dies directly after him in the period 10 July 1400 to 12 September 1400. Actual source: ARB, Rekenkamer, no. 14229.
35. Vandenbroeck, *Extraits analytiques*, I, p. 47. The aldermen enacted a regulation on 17 July 1400 that forbade the ringing of the bells to honor the dead. Idem., pp. 47-8; on 21 July 1400 a procession was announced in the St.-Martinus Church « *pour multiplier et sauver les biens de la terre et preserver le people de l’epidemie et de mort soudaine* ». ‘*Mort soudaine*’ refers to the swiftness of death, and therefore could be a good indication of plague.
36. In 1401/2 ; F. Vinchant, ed., *Annales de la province et comté de Hainaut*, III (Mons, 1848), p. 32.
37. Molhuijsen, ‘Vroegere ongezondheid’, p. 64. In 1398 according to the 18th-century ‘*Kronijk van Deventer*’ by Sef Moonen.
38. T. Hemerken a Kempis, *Opera Omnia*, ed. M.J. Pohl, VII (Freiburg im Breisgau, 1922), p. 373 (1401).
39. M. Pagart d’Hermansart, ed., *Un ordonnance médicale contre la peste vers 1400* (St. Omer, 1901).
40. Vangassen, ‘De pestepidemieën’, p. 29. An increase in the mortmain payments for 1400/1. Actual source: ARB, Rekenkamer, no. 13547.
41. Ibid., p. 30. An increase in the goods of bastards that passed on to the lord after their death. Actual source: ARB, Rekenkamer, no. 13680; also bailiff accounts.
42. Ibid., p. 32. An increase in the mortmain payments for 1400/1. Actual source: ARB, Rekenkamer, no. 13812; also bailiff accounts.
43. Ibid., p. 30. An increase in the goods of bastards that passed on to the lord after their death. Actual source: ARB, Rekenkamer, no. 13925; also bailiff accounts.
44. Large rise in mortalities in the mortmain; ARB, I 004 17870.
45. Bacha, ed., *La chronique liégeoise*, p. 442; “*resurrexerunt flagellatores*” in 1400.
46. J. De Stavelot, *Chronique, 1440-1449*, ed. A. Borgnet (Brussels, 1861), p. 17; high mortality suggested of 12,000 victims in 1401.
47. Ibid., p. 17; 500 mortalities close to Jemeppe.
48. Ibid., p. 17; mentions troubles of the inhabitants of Vivegnis.
49. J. Bijndop, ed., *Kamper Kronijken: De annalibus quaedam nota*, I (Deventer, 1862), p. 2; “*Jnt jair ons heren m.cccc was hier omtrent ouer al jn den lande grote pestilencie*” (1400).
50. P.N. van Doorninck, ed., *Acten betreffende Gelre en Zutphen 1400-1404* (Haarlem, 1901), pp. 28-9; ordinance against the flagellants (4 May 1400).
51. A. van Slichtenhorst, ed., *XIV. Boeken van de Geldersse geschiedenissen. Van ’t begin af vervolghd tot aen de afzweeringh des Konincx van Spanien* (Arnhem, 1654), p 172; *“...verslond de smetziekte veele menschen...Bommel in Gelderland zoud alleen 4000 dooden hebben geteld...*” (1400).
52. H. Frensw., *Het Frensweger handschrift*, eds. W.J. Alberts & A.L. Hulshoff (Groningen, 1958), pp. 166-72; “*Ende desghelijck mitter selver pestilencien stroven in heer Everds hues oerre veel van priesteren, clerken ende leken*” (1401).
53. Ibid., pp. 166-72; all residents of Marienwolde at Frenswegen die one after another (June 1401).
54. F. Nettesheim, *Geschichte der Stadt und des Amtes Geldern* (Crefeld, 1863); found in Stadsarchiv Geldern A15; Willem van Gullik mentions great amounts of death there (26 June 1401).

**1409-11**

| **Region** | **Quantifiable evidence of raised mortality** | **Descriptive mentions of plague in the contemporary sources** | **Descriptive mentions of plague in later sources** |
| --- | --- | --- | --- |
| Na; Lim; Fl; Ho; Br; To; Ze; Gu | Bruges (1); Leiden (2); Breda (3); Gorinchem (4); Middelburg (6) | Maastricht (7); Zutphen (8) | St.-Omer (5); |

1. Blockmans, ‘The social and economic effects of plague’, p. 852.
2. Ladan, *Gezondheidszorg*, p. 234. In 1411 there were 38 dead *fautores*, roughly 50% above the norm – peak mortality in September and October. The St. Catharine’s *gasthuis* received 48 bequests, compared to just over 12 in the general period 1401-20, and there were 70 graves dug in the cemetery compared to just under 29 per year 1401-7 and 1413-20. The *gasthuis* took on extra personnel in the summer of 1411 on account of ‘*doet starf*’.
3. Gooskens, ‘Pestepidemieën’, p. 31. Necrology data.
4. J. Zuijderduijn, ‘Living la vita apostolica. Life expectancy and mortality of nuns in late-medieval Holland’, *CGEH Working Paper Series*, 44 (2013), p. 21.
5. Derville et al., *Histoire de Saint-Omer*, p. 73. The bishop fled to the refuge of Arques at this time to escape the plague.
6. Van den Driest, ‘Hondenbaan’, p. 38; 522 dogs killed between 1407 and 1409.
7. J. Habets, ed., ‘Chronijk der Landen van Overmars’, *Publications de la Société Historique et Archéologique dans le Duché de Limbourg*, 7 (1870), p. 15; mentions “*groet sterfte*” after the city was under siege the year before.
8. RAZ, Arch. Bornhof, 688, no. 89; “*deser die crancken plegen te liggen die an pestilentie tijden*” (1408).

**1413-16**

| **Region** | **Quantifiable evidence of raised mortality** | **Descriptive mentions of plague in the contemporary sources** | **Descriptive mentions of plague in later sources** |
| --- | --- | --- | --- |
| Ar; Fl; Ca; Ho; To; Ha; Ze | Arras (2); Douai (4); Cambrai (5); Gorinchem (6); Middelburg (9); [Hainaut] (10) | Arras (1); Bailiwick of Hesdin (3); Conchy (7) | [Artois] (8) |

1. Archives Municipales d’Arras, BB 5, fo. 37v. The aldermen that came together on 3 October 1413 asked for a tax exemption « *tant pour la mortalité comme pour che que en icelle ville queurt peu ou neant de merchandise* ». Also on 27 June 1416, John the Fearless recalled his subjects from the surroundings of Arras were severely hit by war and disease « *grande et piteuse mortalité d’ypedimie par quoy les laboureurs et peuple… ont esté et sont si vexez, travailliez et diminuez…* », and the mortality lasted two years. Bocquet, *Recherches sur la population rurale*, p. 73. Actual source: ADN, B, no. 13903.
2. ADPdC, Archives Hospitalières d’Arras, 1E 13. The account of the Hospital of St-Jean in Arras for 1414/15 indicates 250 burials. This is an isolated account.
3. Bocquet, *Recherches sur la population rurale*, p. 73. In the account of the Bailiwick of Hesdin there is a patent letter from 28 September 1414, referring to the terms of a pastoral letter of 8 May that gave a discount of 100 pounds to Jean Billet, farmer provost of Hesdin (from 300 pounds). The duke granted this rebate, « *pour cause de la mortalité et pestilence, guerre et aultres causes et consideracions* », and because the situation had worsened since 8 May. Another patent letter from 10 October 1414 reimbursed 200 pounds to Jehan Guerboede, wood merchant for the losses he had suffered « *à cause de la mortalité qui oudit temps a esté ou pays d’Artois et meismement en ladicte ville de Hesdin, comme pour les guerres qui ont esté en icelli pays, pour lesquelles mortalités et guerres plusieurs marchans et aultres dises de la dicte foret n’ont osé ne peu aller ne venir querir lesdictes denrees et marcandises…* ». Actual source: ADN, B, no. 15325.
4. Deregnaucourt, ‘Autour de la mort à Douai’, I, p. 60. Rise in the number of wills.
5. Neveux, ‘La mortalité des pauvres’, p. 79.
6. Zuijderduijn, ‘Living la vita apostolica’, p. 21.
7. Bocquet, *Recherches sur la population rurale*, p. 74. Pierre Evrelenc, Bailiff of Conchy, received a rebate of 40 pounds from the Duke, because merchants did not dare venture into his land. Actual source: ADN, B, no. 15325.
8. J-A. Buchon, *Chroniques d’Enguerrand de Monstrelet : en deux livres, avec pièces justificatives (nouvelle édition)*, II (Paris, 1858), p. 468. It was noted in 1414 by Picardy chronicler Monstrelet that « *régnoit par toutes les parties du royaume de France et en divers pays une maladie qui se tenoit en la tête, de laquelle moururent plusieurs personnes, tant vielz que jeunes ; et nommoit-on ladicte maladie la coqueluche* ».
9. Van den Driest, ‘Hondenbaan’, p. 38; 390 dogs killed between 1415 and 1416.
10. Large rise in mortalities in the mortmain; ADN, B 12177.

**1420-3 (only in Northern Netherlands)**

| **Region** | **Quantifiable evidence of raised mortality** | **Descriptive mentions of plague in the contemporary sources** | **Descriptive mentions of plague in later sources** |
| --- | --- | --- | --- |
| Ho; Fr; Ne; Gu; Ov; Br; Gr; Fl; LvA; | Leiden (1); Breda (11); Gorinchem (12); | Rotterdam (2); Oudewater (4); Utrecht (city and countryside) (5); Arnhem (6); [Guelders] (8); Groningen (10); Zwolle (7); Aalst (13); Grave (14); Kempen (15) | East Fryslan (3); Deventer (9) |

1. Ladan, *Gezondheidszorg*, p. 235. Leiden was under siege in 1419, but evidence suggests a plague epidemic in the follow-up years of 1420/1, where there were 75 buried in the St. Catharine’s *gasthuis* cemetery, more than double the average for the general period 1412-25.
2. H. Moquette, ‘Pestepidemieën in Rotterdam’, *Rotterdamsch Jaarboekjes*, 3.3 (1925), p. 12.
3. C. Schotanus, *Beschryvinge end Chronijk vande heerlickheydt van Frieslandt Tusschen ’t Flie end de Lauwers* (Franeker, 1655), passim.
4. N. Plomp, *Ziekenzorg in Woerden* (Woerden, 1980).
5. Kuys, ed., *De Tielse Kroniek*, no. 769; “*In 1421 heerste er in bijna het gehele bisdom Utrecht zware pest (ook in Gelre was er ernstige pest), zodat in Utrecht op één dag meer dan honderd mensen de dood vonden*”.
6. Benders & Borsch, ‘Samenleving’, p. 159; mentions flight of aldermen due to plague, found in city accounts. Also van Veen, ‘De pest’, p. 4, 8-10; “*ordinieren…omme der pestilencien wille*” (1421).
7. Hemerken a Kempis, *Opera Omnia*, VII, p. 385 (1421/2); “*Eodem anno [1421] in mense Septembri pestilential adhuc graviter invalescente quosdam de nostris familiaribus hic moribus invasit. In octava nativitatis Mariae virginis obii infra summan missam quidam laicus...*”.
8. Kuys, ed., *De Tielse Kroniek*, no. 769; “*In 1421 heerste er in bijna het gehele bisdom Utrecht zware pest (ook in Gelre was er ernstige pest), zodat in Utrecht op één dag meer dan honderd mensen de dood vonden*”.
9. Molhuijsen, ‘Vroegere ongezondheid’, p. 64. In 1421 according to the 18th-century ‘*Kronijk van Deventer*’ by Sef Moonen. Also Hemerken a Kempis, *Opera Omnia*, VII, p. 385
10. F. Bakker and R. Nip, ‘De abdij van Aduard en de Cisterciënzer Orde’, in Mol (ed.), *De abtenkroniek van Aduard*, p. 70.
11. Gooskens, ‘Pestepidemieën’, p. 31. Necrology data.
12. Zuijderduijn, ‘Living la vita apostolica’, p. 21.
13. Ordinance mentioned for 1423 in H. Pleij, *De sneeuwpoppen van 1511. Literatuur en stadscultuur tussen middeleeuwse en moderne tijd* (Amsterdam, 1988), p. 147.
14. van Veen, ‘De pest’, p. 3; “*Item soe hefft meyster Laurens doen maecken yn der apotheken tot Arnhem yn Johan Tolhus huys tot behoeff mijnre gnedige vrouwen tegen die pestilentie ende haeren gnedige gesant tot Grave*” (1419).
15. Hemerken a Kempis, *Opera Omnia*, VII, p. 385; *“…fuit notabilis pestilentia…in mense Septembri pestilential adhuc graviter invalescente*” (1421).

**1426-9**

| **Region** | **Quantifiable evidence of raised mortality** | **Descriptive mentions of plague in the contemporary sources** | **Descriptive mentions of plague in later sources** |
| --- | --- | --- | --- |
| Ho; Br; Ar; Ha; To; Me; Ho; Gu; Fl | Leiden (1); Breda (2); Mechelen (7); [Hainaut] (9); Ghent (14) | St.-Omer (4); Valenciennes (5); Halle (8); Ath (10); Gouda (11); Arnhem (12); Steenderen (13) | Tournai (6); Hoorn (3); |

1. Ladan, *Gezondheidszorg*, p. 235. A modest increase in dead *fautores* (17 in 1426), and was roughly 50% above average. The memory book labels ‘*pest*’ as the cause of much death in January 1426 (1 case) and in October 1426 (many cases).
2. Gooskens, ‘Pestepidemieën’, p. 32. Necrology data.
3. Velius, *Chronyk*, p. 49. First published in 1604.
4. BASO, MS 930, 1, fos 256, t2, 62v, 170; MS 933, fo. 365. AMSO, Registre des délibérations échevinales, B fos 27, 73v, 77; C fo. 56v; B 248.3; B 239.2; 2G 453 fos 5, 40, 44v; 2G 454, fos 7, 32, 115-6, 139v-41, 146v.
5. ADN, 40 H, no. 167; in 1428.
6. On 3 July 1426 a procession was announced; « *pour fair cesser les tres grieves pestilences et mortalités qui soudainement a present de jour en jour sont en icelle ville* ». A. de le Grange, ed., *Extraits analytiques des registres des consaux de la ville de Tournai, 1431-1476* (Tournai, 1893), p. 209.
7. A spike in the necrology of the ‘*geschoeide karmelieten*’, 11 in 1426 and 10 in 1427 when double figures was rare; <http://www.mechelsegenealogischebronnen.be/Databank>>. Also an explicit mention of someone who died ‘fleeing the plague’ in 1426.
8. M. Van Den Weghe, ‘Hallensia III. Besmettelijke ziekten te Halle’, *Gedenkschriften van de K. Geschied- en Oudheidkundige Kring van Halle*, 10 (1934-5), p. 7. There were no masons available to restore the mill on account of the high plague mortality in 1428.
9. Moderate rise in mortalities in the mortmain; ADN, B 12187.
10. E. Fourdin, ed., *Inventaire analytique des archives de la ville d’Ath*, I (Brussels, 1873), p. 15; raising of the ‘*maltôte*’ (special tax on consumption) in June 1428 to compensate for population losses on account of the epidemic of the past years.
11. D.P. Oosterbaan, ed., ‘Kroniek van de Nieuwe kerk te Delft’, *Haarlemsche Bijdragen: Bouwstoffen voor de Geschiedenis van het Bisdom Haarlem*, 65 (1958), p. 109; “*een man was, die die gave Gods hadde...tot Gouda*” (1426).
12. D. De Man, ed., *Hier beginnen sommige stichtige punten van onsen oelden zusteren* (The Hague, 1919), p. 104, 107; “*Hiernae waert si zieck ander pestelenciën*” (1429).
13. GA, Arch. Kerk Steenderen, 1, fo. 1; priest Johan van Walbeck mentions in his parish “*magna viguit pestilentialia*” (1429).
14. SG, Oud Archief, no. 330; there is a spike in inventories after death in 1425-6 of 122 and 149 acts respectfully compared to 77 on average in the previous decade.

**1435-40**

| **Region** | **Quantifiable evidence of raised mortality** | **Descriptive mentions of plague in the contemporary sources** | **Descriptive mentions of plague in later sources** |
| --- | --- | --- | --- |
| Fl; Ar; To; Ca; Ne; Ho; Br; Ha; Li; Na; Ov; LvA; Gu; Dr | Bruges (9); Tournai (11); Cambrai (12) ; Breda (16); [Hainaut] (17); [Liege] (18); [Flanders] (20); Leiden (21); Arras (34); Houdain (35); Vilvoorde (37); Aalst-Geraardsbergen (38); Deinze (39); Courtrai (40); Tielt (41); Molenbeek (42); Puttem (43); Ruislede (44); Wingene (45); Ursel-Wessegem (46); Zomergem (47); Waarschoot (29); Lovendegem (30); Utrecht (32); [Hainaut] (48); Ghent (53) | Zuydcoote (1); [Artois] (2); Eclimeux (6); Ecquermicourt (7); Utrecht (city & countryside) (14); Rotterdam (15); Antwerp (24); Zwolle (25); St.-Omer (36); Ghent (31); Den Haag (22); Delft (49); Culemborg (50); Arnhem (51); Anloo (52) | Auxi-le-Château (3); Aubin (4); Rollepot (5); Aire (8); Bruges (10); Amersfoort (13); [Namur] (19); Ghent (26); Ypres (27); [Artois] (28); Utrecht (33); [Utrecht, Amersfoort, Gelderland, Holland, Zeeland] (23) |

1. Bocquet, *Recherches sur la population rurale*, p. 74. The aldermen of Zuydcoote signalled that in the account of 1435/6, like they had done previously in 1431, the ‘*assis*’ had brought in only a small amount of money « *pour ce que en icelles annee, on y dispensa peu de breuvages pour le grant mortalité quy fut audit lieu et environ en l’année précédente* ». That year the ‘*assis*’ only yielded 34 pounds compared to 54 pounds in 1433.
2. Ibid., p. 74. In 1439 the Duke ordered Martin Cornille, the aid recipient of Artois and Picardy to take 1400 pounds « *obstant les meschiefs, mortalitez, guerres et famines et povreté du peuple des pays* ». Actual source: ADN, B, no. 1967.
3. Ibid., p. 74. In 1441, the Duke gave a tax rebate to Auxi-le-Château, « *par la mortalité qui esté en ladicte ville, plusieurs des residens en icelle sont trepassés et tellement que […] ladicte ville, pour la plus grant partie, est demouree inhabitee et desolee ».* Plague occurred together with war in this locality, and in 1437 the English had burned between 160 and 180 households. Idem., p. 156. Actual source: ADN, B, nos 1973, 17672.
4. Ibid., p. 74. In 1441 the Duke also recalls mortality in Aubin, « *grant famine et povreté a esté, dont très grant mortalité s’est ensuye audit pays et par especial, dans la dicte ville, qui par ce moyen, apres la desolation faite par les Anglois est encore diminuee de peuple et de biens*… ». Actual source: ADN, B, no. 1979.
5. Ibid., p. 74. On 23 October 1443, the seigneur of Rollepot wrote: « *Les habitants de ma dicte terre de Rolepot sont telement diminuez, tant par le fait de guerres, comme par mortalitez et autrement que des piéça, il ne y en a encores quelquepersonne demourant en icelle terre, sy non seulement mon censier* ».
6. Ibid., p. 74. The accounts of the aides signal that in Eclimeux, « *pour la grant mortalité qui a esté en ladicte ledit temps durant [1438-1439], est ladicte ville demouree inhabitee jusques a present* ».
7. Ibid., p. 74. In Ecquemicourt they did not receive any payment for the aides, « *tant pour la povreté des gens et la grant mortalité, comme pour ce que ladicte ville a esté arse par les Angloix*… ».
8. P. Bertin, *Une commune flamande artésienne, Aire-sur-la-Lys, des origines au XVIe siècle* (Arras, 1947), p. 388. Letters from Philip the Good dated October 1439, where he revoked certain customs for the city of Aire. Here we read that « *pour la grant mortalité qui esté en ladicte ville en l’an 1438, ou il y a mort 4000 personnes ou plus, icelle ville est inhabitee et une grant partie des maisons gastees et en grant desolacion* ». This is an over-exaggeration or poor estimate because Aire did not have more than 4000 inhabitants in total.
9. One in three ‘bastards’ had died. The ‘*Bien des Batards*’ was a source that recorded the possessions that came into the hands of the Duke upon their death, found in the Bailiwick accounts. Van Gassen, ‘Pestepidemieen’, pp. 61-70. Actual source: ARB, RE Baljum, no. 13773.
10. Van Dixmude, *Corpus croniconun Flandriae*, III, p. 103. “*In Vlaanderen storven vele lieden van honghere, ende het was al Viaendren duere eene groote staerfte generael van der bootze, van den brande, van hitteghe cortsen ende van der epedemye ; seensdaeghs was tvolc ghezont, tsanderdaeghs siec, sdaerdaegs men begrouft in der eerden ; ende deze staerfte beghonste in de Mey ende gheduerde tot na St. Martinsmesse (11th November 1438)*”*.* Daily 22-24 corpses were counted in four parishes, sometimes 29-30. On 16 August there were 36 victims in one parish and 39 in another; in the first parish they numbered 35 on the 19th and 32 on the 20th. "*Men zeide voorwaer te Brughe omtrent St. Baefsdagh (1st of October) dat dat vijfste deel van den volke van Brugghe was ghediminueert ende ghemindert bij der straeften, dat zonderlinghe vele Heden doot waren, de zomeghe gevloon van aermoede, de zomghe omme de quade neringhe of van sculden de stede van Brugghe ruumden*". SB, Kroniek van Vlaanderen, Handschrift 436, Band A: Band B, no. 193 (f°CCIII v°b); 7 July 1438, “*Item up den zevensten dach van hoymaent, doe was onthooft bij den souverein Pieter Christiaens, deken van den sceppers te Malen. Ende up de zelve tijt zo starf so zeere te Brucghe datter up zommeghe dach waren XXXVI lijcken. Ende binnen den zelven jare zo storven te Brucghe wel XXIIIIM personen van der pestelencie*”.
11. Vangassen, ‘De pestepidemieën’, p. 71. Despite destruction of the archives during the Second World War, Adolphe Hocquet published an alphabetical table of wills, which show a clear rise in 1438 to 328 when most years rarely exceeded 100, and often no more than 50.
12. ADN, 3H273; obituary from Abbaye du Saint-Sepulcre de Cambrai, « *1438 la peste estoit a Cambray* » (1438).
13. W. van Rootselaar, *Amersfoort 777-1580* (Amersfoort, 1878), p. 35.
14. A. van der Weyde, ‘Bijdrage tot de geschiedenis der pest te Utrecht’, *Nederlands Tijdschrift voor Geneeskunde*, 71b (1927), p. 3119. Also K. Burman, ed., *Utrechtse jaarboeken van de vyftiende eeuw, vervattende het merkwaardige in het gesticht...*, I (Utrecht, 1754), no. 512; “*de pest bracht veel sterfte in het Sticht*”. A prayer in protection against the plague from 1440; M.H. Hulshof, ed., ‘Gebed en voorschriften tegen pest in een Utrechtsch getijdenboekje uit 1440’, *Nederlands Tijdschrift voor Geneeskunde*, 83 (1939), pp. 533-5. Also *Kronijk van Arent toe Bocop*, p. 525; “*dat derde iaer daerna [after 1437] quam hyer in den lande een grotte swarre pestelensie und ffort dye Ryn opwers*” (1440).
15. Moquette, *Pestepidemieën*, p. 12.
16. Gooskens, ‘Pestepidemieën’, p. 33. Necrology data.
17. Vangassen, ‘De pestepidemieën’, pp. 30-4.
18. Ibid., pp. 56-70. Also De Stavelot, *Chronique*, 145; « *mortaliteit…par fivres contagieux…chaudes maladies* in 1438 ».
19. Galliot, *Histoire Générale*, V, no. 1438; « *Sous le règne de Philippe-le-Bon, duc de Bourgogne, le comté de Namur, fut affligé par deux grands fléaux, la peste et la famine* ».
20. Vangassen, ‘De pestepidemieën’, pp. 30-4.
21. Ladan, *Gezondheidszorg*, p. 236. The number of benefactors increased in 1439 to 30, when the average over the period 1427-49 was little more than 6. The intensity of mortality peaked in August-October 1439, and the memory book had already noted plague in June and August of that year. The number of testaments also made increased in 1439 (30) compared to 8 in 1440/1 and 12 in 1441/2, and there was a large increase in the number of coffins bought – 55 in 1439 was much higher than that seen in the middle of the 1430s.
22. T.S. Jansma, *Raad en rekenkamer in Holland en Zeeland tijdens hertog Philips van Bourgondië* (Utrecht, 1932), p. 159. Clerk Pieter van der Tannerie died in 1439 of plague in the Den Haag.
23. A. van Bemmel., *Beschrijving der stad Amersfoort*, I (Utrecht, 1760), p. 927; “*1439 Was ‘er t’Utrecht, t’ Amersfoort en in ’t gantsche Stricht, alsmede in Gelderland, Holland, Zeeland en langs de Rhyn een grote Pestilentie onder de menschen, zo dat alhier in die tyd in een klooster aan de besmetting gestorven zyn een en twintig Nonnen*”.
24. RA, Correctie boeck, 1414-1512, fo. 67v. On 13 July 1439, it was noted that Katline van den Berghe was punished “*overmits dat veele haestlinge van der haestiger ziecte, in de stove, die de selve Katline houdt, in der nacht gestorven zyn, en dat zy daer en boven rechtevoert de stove ophielt en stoefde, verbiedende hoeren familien dats niemand en seyde noch wt brachte, twelc emmers was om de gantze stad te punieren en in sterfte te brengen*”.
25. Hemerken a Kempis, *Opera Omnia*, VII, p. 390 (1440).
26. Van Heule, ed., Memorieboek, I, p. 210; “*Item, in dit jaer was te Ghent eene groote sterfte*” (1439).
27. ARB, Rekenkamer, 14548; « *De feu sire perpian Kerayser pauvre bastauqui terspassa en la dite ville dur la seignourie d’aratrique de la malvaise maladie* ».
28. H. van der Linden, ‘Eene reis door de Nederlanden in 1438’, *Société Chorale et Littéraire des Mélophiles de Hasselt*, 46 (1906), p. 45.
29. J. De Smet, ‘Le dénombrement des foyers en Flandre en 1469’, *Bulletin Commission Royale d’Histoire*, 99 (1935), pp. 105-50. Increase in the mortmain payments in the years 1437/8 compared to previous and following years. In 1438 it went up to 38, when it was never more than 3 between 1434 and 1440. Actual source is the ‘*Rekeningen van de baljuw van Oudburg*’.
30. Ibid. Increase in the mortmain payments in the years 1437/8 compared to previous and following years. Actual source is the ‘*Rekeningen van de baljuw van Oudburg*’.
31. Vangassen, ‘De pestepidemieën’, p. 58. The aldermen of Ninove were called to an assembly of the ‘*Raad van Vlaanderen*’ in Ghent, but did not go because they wanted to avoid plague raging there in 1437/8; “*Mits der sterften ende andersins dat mijn voors heeren van den Rade hen absent hilden van der Cameren langhen spacie van tide, te wetene tusschen der impetracie der voors lettren en den 6en dach van novembre*”. Actual source: ARB, Rekenkamer, Stadsrekening Ninove 1437-8.
32. Only two residents remained in the Regulierenklooster (monastery) in Utrecht after the plague of 1439; C. Block, ‘Kroniek van het Regulierenklooster te Utrecht’, *Bijdragen en Mededelingen van het Historisch Genootschap*, 16 (1895), p. 58.
33. L.G. Visscher, ‘Oorspronck, beginsel ende voortganck, voorders wat Fundateurs, Overste ende Regeerders dattet convent int Geyn buyten Utrecht gehadt heeft, byeenvergadert door Io. Buysling, pater aldaer, anno 1574’, *Tijdschrift voor Geschiedenis, Oudheden en Statistiek van Utrecht*, 8 (1842), p. 186.
34. The accounts of the hospital St.-Jean in Arras form a continuous series starting from 1437/8. In that year they buried 295 bodies, 802 in 1438/9 and 149 in 1439/40. In 1440/1, the situation normalized and they did not dig more than 46 pits. ADPdC, Archives Hospitalières d’Arras, 1 E, no. 16; and following years. From 1 E no. 21 (1442-3) all of the accounts for the fifteenth century have been burnt, and their previous existence is known through an inventory published in 1914.
35. The accounts for the domain of Houdain include a section on the rights of the bourgeoisie, where each burgher paid 4 deniers. The accounts are accompanied by lists of those included for years 1436/7 and 1443/4. In 1436/7, 72 burghers paid, though we only have names for 42 because the list is damaged. In 1443/4, the largest part of these names do not correspond with those from the 1436/7 account, and some lands were abandoned after the death of owners. There were only 44 bourgeois subject to the levy. These numbers, together with defaults in payments, suggest that the plague of 1438 had significant demographic impact here; ADN, B, nos 14482-15484.
36. Derville, ‘La population’, p. 67. Actual sources: BASO, MS 930, 1, fos 256, t2, 62v, 170; MS 933, fo. 365. AMSO, Registre des délibérations échevinales, B fos 27, 73v, 77; C fo. 56v; B 248.3; B 239.2; 2G 453 fos 5, 40, 44v; 2G 454, fos 7, 32, 115-6, 139v-41, 146v.
37. J. Cuvelier, ed., *Les dénombrements de foyers en Brabant (XIVe-XVIe siècle)* (Brussels, 1912), no. CXLIX. Cuvelier refers to the plague outbreak of 1438 which caused a decline of 130 households compared to the numbers of 1437.
38. Vangassen, ‘De pestepidemieën’, p. 29. Increase in the mortmain payments between September 1437 and September 1438 compared to previous and following years. Actual source: ARB, Rekenkamer, no. 13547.
39. Ibid., p. 30. Increase in the mortmain payments in the years 1438/9 compared to previous and following years. Actual source: ARB, Rekenkamer, no. 13901.
40. Ibid., p. 32. Increase in the mortmain payments in the years 1438/9 compared to previous and following years. Actual source: ARB, Rekenkamer, no. 13816.
41. Ibid., p. 33. Increase in the mortmain payments in the years 1437/8 compared to previous and following years. Actual source: ARB, Rekenkamer, no. 13816. Also noted « *Nommen son trespassex hastement de l’impedemie* ».
42. Ibid., p. 33. Increase in the mortmain payments in the years 1437/8 compared to previous and following years. Actual source: ARB, Rekenkamer, no. 13816. Also noted « *Nommen son trespassex hastement de l’impedemie* ».
43. Ibid., p. 33. Increase in the mortmain payments in the years 1437/8 compared to previous and following years. Actual source: ARB, Rekenkamer, no. 13816. Also noted « *Nommen son trespassex hastement de l’impedemie* ».
44. Ibid., p. 33. Increase in the mortmain payments in the years 1437/8 compared to previous and following years. Actual source: ARB, Rekenkamer, no. 13816. Also noted « *Nommen son trespassex hastement de l’impedemie* ».
45. Ibid., p. 33. Increase in the mortmain payments in the years 1437/8 compared to previous and following years. Actual source: ARB, Rekenkamer, no. 13816. Also noted « *Nommen son trespassex hastement de l’impedemie* ».
46. Ibid., p. 34. Increase in the mortmain payments in the years 1437/8 compared to previous and following years. Actual source: ARB, Rekenkamer, no. 14455.
47. De Smet, ‘Le dénombrement des foyers’. Increase in mortmain payments in the years 1437/8 compared to previous and following years. In 1438 it was 17, when it never exceeded three between 1434 and 1440. Actual source is the ‘*Rekeningen van de baljuw van Oudburg*’.
48. Large rise in mortalities in the mortmain; ADN, B 12205-6.
49. Oosterbaan, ed., ‘Kroniek’, p. 206, 208; “*Anno XXXIX was die grote sterft...*” (1438).
50. A. van de Ven, ed., *Het oud-archief van de gemeente Culemborg* (Utrecht, 1938), no. 187; stadsrekening 1438/9 “*van dat sij die wake snachts verwairt hebben inder starften dat men doe quellic waicte*”; Also idem, no. 188; stadsrekening 1439/40 “*Broeder Elyas, soe hy in der sterften in onss alre nooit bystont*” (1440).
51. GA, Oud Archief Arnhem, Stadsrekeningen, 1244, 1439/40, fos 11, 35, 44; “*burgermeisters, scepenen ende rade ende waren weder inkom die meiste deel die uyt geweest waren omme der pestilencie...want men gheen baden gekrijgen en conde ende oick want hy omme der pestilencie wille, daer quellicken by een omme gekrigen conden...ende waren the samen alle dieghene die vaste buyten der stat om der pestilencie wille geweest waren*”.
52. F. Keverling Buisman, ed., *Ordelen van de Etstoel van Drenthe 1450-1504 (1518)* (Zutphen, 1994), p. 60, 62-3 (August 1441).
53. SG, Oud Archief, no. 330; a spike in inventories after death in 1439 of 211 acts compared to 71 on average in the previous decade.

**1450-9**

| **Region** | **Quantifiable evidence of raised mortality** | **Descriptive mentions of plague in the contemporary sources** | **Descriptive mentions of plague in later sources** |
| --- | --- | --- | --- |
| Ca; Ar; Ho; Ne; Ov; Gu; Fl; Br; Ha; LvA; Na; Bo | Cambrai (1); Leiden (3); Bruges (8); Brussels (9); Mons (10); Breda (13); Oudenaarde (17); Louvain (30); Tienen (31); Landen (32); Aerschot (24); Sichem (25); | Dordrecht (4); Utrecht (5); Zwolle (6); Monnickendam (11); Rotterdam (12); IJsselstein (18); [Brabant] (20); Kampen (22); Antwerp (26); St.-Omer (28); [Artois] (29); Arras (7); Hoorn (16); Hasselt (14); [Gelre] (34); Rosendaal (35); Venlo (36); Zutphen (37); Deventer (38); Arnhem (42); Oisterwijk (43); [Holland] (44) | Lens (2); Amersfoort (19); Montreuil (23); Namur (21); Deventer (15); Douai (27); Zutphen (33) ; Harderwijck (39); Gouda (40); Diepenveen (41); |

1. Neveux, ‘La mortalité’, 78.
2. Bocquet, *Recherches sur la population rurale*, p. 76. In 1456 the duke was advised to reimburse the inhabitants of Lens their part of the aid funds. This city was seen as a fortress of the highest importance, and so it was deemed appropriate that after the epidemic that « *le peuple qui y est de present n’ait cause de l’abandonner de en départir, mais que ceulx qui à l’occasion de la dicte pestilence s’en sont éloignez…aient cause de y retourner. De present, pour cause de la mortalité qui derrenièrement a esté en icelle ville, n’y sont demeurez que environ quatre vins feux au plus, povres gens vivans de labour* ». Actual source: ADN, B, no. 17679.
3. Ladan, *Gezondheidszorg*, p. 236. The number of benefactors was 20% above the norm in 1449, 15 *fautores* died in 1450, against an average of 4 between the years 1440-66. The memory book mentioned in 1450 plague as cause of much death in June, October and November 1450.
4. J. Fruin, ed., *De oudste rechten der stad Dordrecht en van het Baljuwschap van Zuidholland* (The Hague, 1882), p. 294; plague ordinances announced in 1450, 1452 and 1458; also confirmed in J.L. Van Dalen, ‘Oude maatregelen in Dordrecht tegen de pest’, *Vragen van den Dag*, 15 (1900), p. 127.
5. Van der Weyde, ‘Bijdrage tot de geschiedenis der pest’, p. 3119 (for 1450). Also (1450) Burman, ed., *Utrechtse jaarboeken*, I, no. 180; “*In den zomer van dit jaar heft de pestziekte veele menschen in deeze Stadt het leven benomen*”; describes measures taken by city: prohibited to exit the city, prohibition on the sale of consumer goods from infected households. Corroborated by N. van der Monde, ‘Pestziekte’, *Tijdschrift voor Geschiedenis, Oudheden, en Statistiek van Utrecht*, 2 (1836), p. 151; city council decide that it was not permissable for aldermen to stay more than eight days outside the city. Also later (1455); Burman, ed., *Utrechtse jaarboeken*, I, no. 180; “*door de pest veel inwoners van Utrecht overleden. Velen trekken buiten de stad om aan besmetting te ontkomen*”; found again in HUA, Stadsbestuur, 1, no. 16.
6. Hemerken a Kempis, *Opera Omnia*, VII, p. 411 (1450 & 1453). Also in 1458, Gerardus Tydemanni was sent to Zwolle to help the people of regulierenconvent of Bethlehem which was struck by a terrible plague; Pohl, *Opera Omnia*, VII, pp. 442-3.
7. Bocquet, *Recherches sur la population rurale*, p. 76. Plague mentioned in Arras in 1454.
8. Blockmans, ‘The social and economic effects of plague’, p. 853. Also RtB, Bishopric Archives, Acta Capitularia Sancta Donatiani; the canons of the St.-Donatian were allowed to leave the town on 17 August 1458 “for fear of the plague which was in several places in the vicinity of Bruges”.
9. Ibid., p. 853.
10. Ibid., p. 853.
11. Two plague deaths mentioned in the Galilea Minor monastery in 1451; J. Besteman and H. Heidinga, *Het klooster Galilea Minor bij Monnickendam* (Dordrecht, 1975).
12. Moquette, *Pestepidemieën*, p. 12.
13. Gooskens, ‘Pestepidemieën’, p. 33.
14. Ordinance of 1450 noted in J. Benders, ‘Demografie van de stad Hasselt (Ov.) tot 1535’, *Overijsselse Historische Bijdragen*, 109 (1994), p. 10.
15. Molhuijsen, ‘Vroegere ongezondheid’, p. 64. In 1458 according to the 18th-century ‘*Kronijk van Deventer*’ by Sef Moonen.
16. J. Steendijk-Kuypers, *Volksgezondheidszorg in de 16e en 17e eeuw te Hoorn: een bijdrage tot de beeldvorming van sociaal-geneeskundige structuren in een stedelijke samenleving* (Rotterdamp., 1994), 166. Plague of 1452: Philip of Burgundy declared to Holland and West-Frisia that he would not come to collect his taxes because of the raging epidemic: original source found in Oud Archief Hoorn, no. 118, fo. 14.
17. Thoen, *Landbouwekonomie*, pp. 1141-53.
18. B. Heesters, ‘O.L. Vrouwenberg te IJsselstein’, *Historische Kring Ijsselstein*, 22 (1982), pp. 42-8.
19. Van Rootselaar, *Amersfoort*, p. 35.
20. R. Van Uytven, ‘Politiek en economie: de crisis der late XVe eeuw in de Nederlanden’, *Revue Belge de Philologie et d’Histoire*, 53 (1975), pp. 1100-1.
21. Galliot, *Histoire Générale*, no. 1455 « *Une maladie contagieuse fit encore de nouveaux ravages, cette année, à Namur, où en mlins de six mois, elle emporta jusqu’à deux milles quatre cent personnes* ».
22. W. ten Kate, ‘De pestkeuren te Kampen’, *Nederlands Tijdschrift voor Geneeskunde*, 66 (1922), p. 1555.
23. Bocquet, *Recherches sur la population rurale*, p. 76. In 1459, the inhabitants of Montreuil reveal « *la povreté et les affaires d’icelle ville et la ruyne de la fortification d’icelle et aussi les mortalitez qui ont esté et continue par longtemps, par quoy le siege de la justice, qui y est la principal membre de nostre bailliaige d’Amiens, y a vacqué* ». Actual source: ADN, B, no. 2035.
24. Cuvelier, ed., *Les dénombrements des foyers*, nos CXLV-CXLVI. Clear decline in population from hearth counts of 1437 to 1464, and explicit mention of plague outbreak in this region in 1458.
25. Ibid., nos CXLV-CXLVI. Clear decline in population from hearth counts of 1437 to 1464, and explicit mention of plague outbreak in this region in 1458.
26. A. Van Schevensteen, ‘Over pestepidemiën te Antwerpen in vroeger tijden’, *Verslagen en Mededelingen van de Koninklijke Vlaamse Academie voor Taal- en Letterkunde* (1932), pp. 1056-7. Ordinance from 1454 states the closure of trading shops for fear of contagious disease.
27. In 1457, Jacques du Clercq mentions an epidemic « *en plusieurs villes et villages, très fort espéciallement à Amiens, Compiègne, Noyon, Douai et ailleur*s ». J. Du Clerq, ‘Mémoires’, in H. Herluison, ed., *Choix des chroniques et mémoires sur l’histoire de France* (Paris, 1875 [1838]).
28. BASO, MS 930, 1, fos 256, t2, 62v, 170; MS 933, fo. 365. AMSO, Registre des délibérations échevinales, B fos 27, 73v, 77; C fo. 56v; B 248.3; B 239.2; 2G 453 fos 5, 40, 44v; 2G 454, fos 7, 32, 115-6, 139v-41, 146v. Also on 15 and 24 January 1456 the aldermen forbade the second-hand clothes dealers to sell the possessions of the dead, « *pour ce que ancore ne avoit eu forte gelee ne souffisant pour avoir purgié le malvais aer mais avoit le temps esté moyte »*. On 19 February, because there had long been no deaths, the second-hand clothes dealers were allowed to sell their products again but under regulations; AMSO, Registre aux déliberations échevinales, B, fo 27. In 1452 and 1454 there was mention of ‘*maladies dangereuses*’; Bocquet, *Recherches sur la population rurale*, p. 76.
29. A letter from the Chambre des Comptes of Lille on 12 March 1454 stated that the pilgrimage of Boulogne had to be delayed because of the war in Flanders and that mortality prevailing in the region had stopped many notables and their servants from coming. ADN, B, no. 17676.
30. Cuvelier, ed., *Les dénombrements des foyers*, nos CXLV-CXLVI. Clear decline in population from hearth counts of 1437 to 1464, and explicit mention of plague outbreak in this region in 1458; anon., *Annales Parchenses, MGH SS XVI* (Hannover, 1869), p. 608; “*Eodem etam anno regnavit pestilentia magna in eadem civitate*”.
31. Ibid., nos CXLV-CXLVI. Clear decline in population from hearth counts of 1437 to 1464, and explicit mention of plague outbreak in this region in 1458.
32. Ibid., nos CXLV-CXLVI. Clear decline in population from hearth counts of 1437 to 1464, and explicit mention of plague outbreak in this region in 1458.
33. RAZ, Inventaris van de collectie Johannes Gimberg (1850-1930), 0142, no. 68, fo. 1; chaplain said to have died from plague in 1458.
34. GA, Hertogelijk Archief, no. 272, fo. 167; plague in the accounts kept by Arnold van Goer, ‘*landrentmeester*’of Gelre (1449/50); *“[…]so des boschmeisters wyff vandenpestilencien syeck was, ende der huecke nyet en hebbe derven toe tasten dan alst wat voirder kompt…*”. Again for the accounts 1450-2, wife of the ‘*bosmeester*’ “*ander pestilencien sieck lach*”; idem., no. 274, fo. 1. Also idem., no. 738, fo.35v-36; accounts of the tollenaar Lobith; “*so et pestilentie was” and “so die tijt sterflich was ende die pestilentie seer regneerde gekoft vur siecken luden ende tot preservativen ende is onder den siecken vast gedeult in affwesen mijnr […] pestilencienpolver*”.
35. GA, Rekening Korenmeester 1439-50, no. 429, fo. 90v; three children of Willem Nollen died of ‘*pestilentia*’ in 1449/50 at Roosendaal.
36. GA, Hertogelijk Archief, Rekening Rentmeester Venlo 1450/51, no.1555, fo. 4v; plague mention. Also GV, Oud-archief Venlo, no. 1194, Stadsrekening I, fos 15-18; plague mentions.
37. RAZ, Oud Archief Zutphen, 0001, no. 1044, overrentmeesterrekening 1458/59; the week before 15 August 1458, wine donated to the chaplain for three plague processions.
38. HCOvD, Oud Archief Deventer MA, no. 130, Cameraarsrekening 1458, fos 7, 14; “*int leste vander sterfte doe weren hyr twee observanten gecomen alse die prior van Zutphen mit ene brueder ons te prediken…*”and “*van Steven Kistemaker hondert ende 66 doetvaete soe cleyn ende groet die luyden omme gads willen inder sterfte gegeven worden…*”.
39. J.T. de Voecht, *Narratio de inchoatione domus clericum in Zwollis. De kroniek van het fraterhuis te Zwolle*, ed. M. Schoengen (Amsterdam, 1908), p. 118; “*in Harderwijck, ubi nonnichil grassabatur pestilentia...*” (1450).
40. G. Kooijman, ed., *Kroniek van Gouda: 1250 jaar Goudse geschiedenis in jaartallen* (Gouda, 1984), p. 28; “*Dominus Theodricus Hoern, tactus pestilencia, obijt...*” (1451); other friars also infected (p. 31).
41. Anon., ‘Van den doechden der vuriger ende stichtiger susteren van Diepen Veen’, <<http://www.dbnl.org/tekst/_van005vand02_01/>> (1904), p. 155, 263, 305, 308-9; on 28 Decemeber 1452, the last nun of the convent died of plague.
42. van Veen, ‘De pest’, p. 5, 38-9; mention of plague in city accounts (1455); “*om der sterftt wille eyn wijle tijts van hyn mytter woeyngen te well getaigt was*” and “*om der sterffte wil die lude oir kynder te huyss hileden ende der schoelre voill gestorven sijn ende die schoelmeister dairomme ledich heeft moeten gaen lange tijt*”.
43. RAT, Oud Rechterlijk Archief Oisterwijk, no. 157, fo. 8 [1449]; act from the magistrates noting ‘*Godsgave*’ and ‘*haestigen dood*’ in Tilburg where the wife of Claes Jan Maes, her 2 children, her parents, and the maid all were victims.
44. NATH, Oud Archief Stadsbestuur Delft, no. 1691; Duke of Burgundy held up in Holland through plague (1452).

**1467-73**

| **Region** | **Quantifiable evidence of raised mortality** | **Descriptive mentions of plague in the contemporary sources** | **Descriptive mentions of plague in later sources** |
| --- | --- | --- | --- |
| Ho; Ne; Ov; Gu; UG; Ze; Fl; Br; Fr; Lim; Ar; Ha; PoC; Li | Leiden (1); Doesburg (9); Bruges (15); Breda (16); Gorinchem (23); Douai (25); Arras (26) | Rotterdam (2); Utrecht (6); Kampen (8); Amsterdam (11); Venray (13); Diest (14); Aduard (17); Antwerp (21); Courtrai (22); Louvain (24); St.-Omer (27); Bergen-op-Zoom (18); Cassel (19); Haarlem (20); Alkmaar (3); Delft (4); Schiedam (5); Dordrecht (12); Bruges (28); Ypres (29); Veurne (30); Wervik (31); Oudenburg (32); Calais (33); Liege (35); [Liege city and countryside] (36); Mons (37); Kampen (38); Montfort (39); Roermond (40); [Overkwartier] (41); Buren (42); | Amersfoort (7); Zierikzee (10); Zutphen (34); Zwolle (44) |

1. Ladan, *Gezondheidszorg*, p. 59. Also Adam van Cleve was required in 1468 to investigate the parts of Leiden which were afflicted by plague; NATH, Archief Grafelijksheidsrekenkamer, Rekeningen, no. 169, fo. 70v.
2. H. Brunner, ‘Pest-epidemieën van de 15^e^ tot de 17^e^ eeuw te Rotterdam’, *Nederlands Tijdschrift voor Geneeskunde*, 90.2 (1946), p. 620; ordinance evidence from 1467.
3. For Alkmaar, NATH, Archief Grafelijksheidsrekenkamer, Rekeningen, no. 169, fo. 70v.
4. Jan Lodijk was required in 1468 to investigate parts of Delft afflicted by plague; M. Damen, *De staat van dienst: de gewestelijke ambtenaren van Holland en Zeeland in de Bourgondische periode (1425-1482)* (Hilversum 2010), p. 118.
5. For Schiedam, Ibid.
6. Van der Weyde, ‘Bijdrage tot de geschiedenis der pest’, p. 3120. Also Burman, ed., *Utrechtse jaarboeken*, I, no. 502; “*de pest vergt weer veel slachtoffers in de stad Utrecht*”.
7. Van Rootselaar, *Amersfoort,* p. 35.
8. Ten Kate, ‘De pestkeuren’, p. 1655. Also Bijndop, ed., *Kamper Kronijken*, 1, p. 17; “*Int jair ons heren m cccc lxviii was hier in der stat eene groete pestilentie, durende van St. Petersdach ad Vinoula thent Alrehwilligen dach off dairomtrent*” (1468).
9. A. Weiler, ed., *Necrologie, kroniek en cartularium c.a. van het Fraterhuis te Doesburg (1432-1559)* (Leiden, 1974), p. xvii. Also GA, Hertogelijk Archief, no. 512, rekening van Johan van Holthuysen rentmeester van der Schuilenburgse goederen, 1467/8, fo. 6; “*alsoe id bynnen Doisborch began to starffen en wolden Johan Hoen, Arnt van Huysen ind die andere rutere nytt langer dair blyven, so byn ick mit de selven aldair oppgebraken ind to Doetincjem gereden des guesdaigen na onser vrouwen dach nativitatis*” (9 September 1467).
10. G. De Moor, *Verborgen en geborgen: het Cisterciënzerinnenklooster Leeuwenhorst in de Noordwijkse regio (1261-1574)* (Hilversum, 1994), p. 200.
11. Adam van Cleve was required in 1468 to investigate the parts of Amsterdam which were afflicted by plague; NATH, Archief Grafelijksheidsrekenkamer, Rekeningen, no. 169, fo. 70v. Also plague ordinance announced in 1471; J. Breen, ed., *Rechtsbronnen der stad Amsterdam* (The Hague 1902), pp. 79-80.
12. Jan Lodijk was required in 1468 to investigate parts of Dordrecht afflicted by plague; Damen, *De staat van dienst*, p. 118. Also Van Dalen, ed., ‘Oude maatregelen’, p. 127; plague ordinance in 1469.
13. M. Flokstra, ‘Pest-epidemie in Venray 1469’, *Jaarboekje met Jaarverslag*, 2 (1984), pp. 1-2.
14. C. De Backer, ‘Maatregelen tegen de pest te Diest in de vijftiende en zestiende eeuw’, in *De Pest,* p. 284.
15. Blockmans, ‘The social and economic effects of plague’, p. 853. The *Acta Capitularia S. Donatiani* of Bruges mention cases of mortality by the plague in 1469 and 1471; SB, Bisschoppelijk Archief, *Acta Capitularia S. Donatiani.*
16. Gooskens, ‘Pestepidemieën’, p. 33. Necrology data.
17. Mol, ed., *De abtenkroniek van Aduard.*
18. Plague ordinance of 27 June 1471; WBA, Stedelijk Archief van Bergen op Zoom tot 1810, no. 8, fos. 71.
19. A letter from the Duke of Burgundy (1472) to the nuns of the hospital of Cassel mentions that plague was present in Cassel in 1471: « *et en temps de pestilence ou autres maladies contagieuses y recevoir et garder tous pauvres malades de notre dite ville et semblablement lesdits passans pelerins et autres miserables personnes illec surprises et demeurans gisans devant leur gisines et faire enterrer les mors trepassez audit hospital dont ils y ont eu l'année passée plus de cent cinquante personnes qui tous y moururent de ladite pestilence ou demaladies contagieuses* ». P.J.E. De Smyttère, *Topographie, historique, physique, statistique et médicale de la ville et des environs de Cassel* (Paris, 1828), pp. 39-41.
20. Adam van Cleve was required in 1468 to investigate the parts of Haarlem which were afflicted by plague; NATH, Archief Grafelijksheidsrekenkamer, Rekeningen, no. 169, fo. 70v.
21. Van Schevensteen, ‘Over pestepidemiën’, p. 1057. New ordinance on 4 April 1472, urging plague sufferers to hold the white stick in public, and mark their infected houses with a bundle of straw accordingly.
22. RK, Oud Stadsarchief Kortrijk, 101/3, nos 5319.
23. Zuijderduijn, ‘Living la vita apostolica’, p. 21. Also Jan Lodijk was required in 1468 to investigate the parts of Gorinchem which were afflicted by plague; Damen, *De staat van dienst*, p. 118.
24. L. Torfs, *Fastes des calamités publiques survenues dans les Pays-Bas*, I (Tournai, 1859), p. 60; E. van Even, ed., ‘Reglement over de pest, afgekondigddoor het stedelyk bestuer van Leuven, anno 1473’, *Vaderlandsch Museum voor Nederduitsche Letterkunde, Oudkunde en Geschiedenis*, 2 (1858), pp. 132-5. Ordinance announced by the Louvain magistrates on 12 April 1473. Another ordinance in 1474; A. Huttmann, A., ‘Ordonnances municipales antipesteuses provenant de la région entre Rhin et Meuse et la Belgique actuelle’, *Histoire des Sciences Médicales*, 17.1 (1982), p. 133.
25. Significant rise in the number of wills; Deregnaucourt, ‘Autour de la mort à Douai’.
26. The accounts for the hospital of St.-Jean in Arras show a prolonged and severe epidemic from 1466/7 until 1471 based on the number of graves that were dug; In 1465/6 (25), in 1466/7 (96), 1467/8 (346), 1468/9 (176), 1469/70 (122), 1470/1 (174), 1471/2 (158), 1472-3 (64). Bocquet, *Recherches sur la population rurale*, p. 77. Original source Archives Hospitalières d’Arras, 1 E, no. 16 and following years.
27. BASO, MS 930, 1, fos 256, t2, 62v, 170; MS 933, fo. 365. AMSO, Registre des délibérations échevinales, B fos 27, 73v, 77; C fo. 56v; B 248.3; B 239.2; 2G 453 fos 5, 40, 44v; 2G 454, fos 7, 32, 115-6, 139v-41, 146v. The city also opened a new cemetery in 1470, “*pour les pestiférés*”; Derville et al., *Histoire de Saint-Omer*, p. 84.
28. Comments on ‘*reeuwerij*’ in 1468 (workers charged with employment in plague-infected houses) and their potential role in poisoning people; J. Huyghebaert, ‘Reeuwers in Vlaanderen in 1468’, *Biekorf,* 68 (1967), p. 98.
29. Ibid.
30. Ibid.
31. Ibid.
32. Archives mention substances found at a house of a known ‘*reeuwer*’ that suggested his guilt (of attempting poisoning) in 1468; E. Feys and D. Van de Casteele, *Histoire d’Oudenbourg*, II (Bruges, 1873), p. 444.
33. ELO, Archief der Secretarie van de stad Leiden, Stadsbestuur 1, no. 1069, reg. 797; *“...daar thans te Calis hevig de pest woedt*”.
34. RAZ, Inventaris van de collectie Johannes Gimberg (1850-1930), 0142, no. 68, fo. 1; plague said to have raged in 1468 – reference taken originally from city accounts.
35. A. D’Oudenbosch, *Chronique* (Liege, 1902), p. 230; mentions ‘*pestilentia*’ in 1470.
36. Habets, ed., ‘Chronijk’, p. 43; “*grote sterft…als van den louppenden buyck ovel in den buyck*” in 1473.
37. ACM, Registre de résolutions du Conseil, no. 1297, fo. 40; “*pluisieurs des mannans de Mons pour cause de la pestilence se departoient de le ville et se alloient tenir a villaige*” (6/8/1468).
38. SK, RA 8, digestum vetus 1448-1478, fo. 79; butchers, bakers and brewers who had a plague victim in the household could not resume their craft until one month after plague had subsided from their household.
39. GA, Hertogelijk Archief, no. 1671, schattingsregister Land van Montfort, sub Odoliënberg, fos 12v-13; lack of payment due to plague mortality (1468); “*Item tot Momfoirt op sente peters avont ad vincula die schattinge van Berge, Postert ind Vlodorp gesat soe die pestenlentie inden dorpen seer was avermytz, den drosset scholtet schepen ind schatmeisters verteert in geritz huyss...*” and *“...tot ruremunde in lenart koxs huys soe die van Berge Postert Lynne Vlodorp indo ick deels van Swalmen oir schattinge dair betailden so idt se opter vurs dorpen starff vander pestilentie…*”.
40. *Ibid.*
41. GA, Hertogelijk Archief, no. 1602, rekeningen Overkwartier, fos 39-40; plague mention.
42. GA, Hertogelijk Archief, no. 339, rekening rentmeester Buren en Beusichem 1469/70, fo. 38; retrospectively (thus 1468), “*also dan te Beuren wall die vierde mensche ghestorven was*” – one in four had died in Buren.
43. Bijndop, ed., *Kamper Kronijken*, 1, p. 17; “*In den selven jare quemen die van Deventer hier liggend voir die pestilencie in der tijt regnierde toe Deventer*” (1467).
44. de Voecht, *Narratio*, p. 150; “*tempore pestis*” (August 1472).

**1481-4**

| **Region** | **Quantifiable evidence of raised mortality** | **Descriptive mentions of plague in the contemporary sources** | **Descriptive mentions of plague in later sources** |
| --- | --- | --- | --- |
| Ho; Fl; Ha; Li; Lim; Na; Fr; Gu; Ov; Br; Ne; LvA; Ar; Ze | [Flanders] (3); [Hainaut] (4); [Liege] (4); Maastricht (5); [Namur] (6); Leiden (8); Dudzele (20); Middelburg (11); Zutphen (14); Kampen (1) | Gorinchem (2); Amsterdam (9); Groningen (10); Culemborg (15); Utrecht (16); Heesbeen (17); Diest (18); Antwerp (19); Aalst (13); Turnhout (7); Dordrecht (21); Arnhem (22); Delft (23) | Deventer (12); Zandwijk (24) |

1. Eighteen councillors died in Kampen in 1483/4; V. Robijn, ‘Brothers in life and death. Religious and social aspects of the Kampen ‘*schepenmemorie*’ (1311-c.1580)’, in H. Brand, ed., *Trade, diplomacy and cultural exchange: continuity and change in the North Sea area and the Baltic, c.1350-1750* (Hilversum, 2005), p. 180. Also Ten Kate, ‘De pestkeuren’, p. 1655; plague ordinances in August and November 1483.
2. M. van Andel, ‘Pestepidemieën te Gorinchem’, *Nederlands Tijdschrift voor Geneeskunde*, 57 (1913), pp. 1844-62.
3. Blockmans, ‘The social and economic effects of plague’, p. 854.
4. Ibid., p. 854.
5. Ubach and Evers, eds., *Historisch encyclopedie*, pp. 409-10.
6. Blockmans, ‘The social and economic effects of plague’, p. 854.
7. E. Adriaensen, ‘Turnhout in het verleden’, <<http://www.stadsarchiefturnhout.be/turnhout-in-jaartallen>> (catalogue of manuscripts in Turnhout City Archive); the sheriff’s accounts mention a ‘*febris pestilencialis*’ – a pestilential fever in 1480.
8. Ladan, *Gezondheidszorg*, p. 59.
9. Plague ordinance announced in 1483; Breen, ed., *Rechtsbronnen*, pp. 79-80.
10. F. Huisman, *Stadsbelang en standsbesef. Gezondheidszorg en medisch beroep in Groningen, 1500-1730* (Rotterdam, 1992), p. 22.
11. Van den Driest, ‘Hondenbaan’, p. 38; 684 dogs killed in 1483.
12. Molhuijsen, ‘Vroegere ongezondheid’, p. 64. In 1483 according to the 18th-century ‘*Kronijk van Deventer*’ by Sef Moonen; “*…was soe groote en sware pestilencie binnen Deventer, dat die een vrunt van den anderen liepen ende nyet by malkanderen dorsten blyven*”. Also the rector and 7 sisters of the Lamme van Diesehuis die through a sickness in 1483, prompting the compilation of the so-called ‘Sisters Book’; O.A. Spitzen, ed., ‘Het leven der eerwaardige moeder Andries Yserens, overste van het Lammenshuis te Deventer, overladen in den jare 1502’, *Archief voor de Geschiedenis van het Aartsbisdom Utrecht*, 2 (1875), pp. 189-216. Also HCOvD, Oud Archief Deventer, Cameraarsrekening 1483, I, fos 3-4; mentions mortality on 27 July, 11 August, 5 October and 11 October; also in Idem., II, mentions plague on 18 August; “*scepene ende raet verbaet ten Diepenvene om der pestilencien willen…*”.
13. ARB, Rekeningen Aalst, no. 31480, fo. 27v; “*Willeme Den Nokere es gheghevene gheweest over zinen arbeyt van dat hy ontrent St. Andriesmesse lestleden als de pestilencie tAelst begonste te cesseerne ende de goede mannen vanden wet ende andere die uut der stede ghevloden waren weder begonsten in te commene tcuyschte, vierde ende verluchtende tscepenhuus boven ende beneden omme alle corruptie te weeren*” (1485).
14. R. van Schaîk, ‘Zutphense geschiedenis: van de elfde tot het einde van de zestiende eeuw’, in W. Frijhoff, B. Looper and J. van der Kluit, eds., *Geschiedenis van Zutphen* (Zutphen, 1989), p. 66; from 1483 to 1489, a necrology shows that 61 new brothers entered the Fraternity of Aldermen, likely linked to the epidemic known for 1483/4.
15. M. van Malenstein, ‘Het Sint-Pietersgasthuis te Culemborg: een archief-studie vanuit medisch-historisch oogpunt‘, *Gewina: Tijdschrift voor de Geschidenis van Geneeskunde, Natuurwetenschappen, Wiskunde en Techniek*, 6 (1982), p. 72. Also van de Ven, ed., *Het oud-archief*, no. 1277, Rekening St. Petersgasthuis Culemborg; raised expenses for purchase of coffins in 1482/3. Also Idem., no. 231; “*dat sy twee goede mannen huerden in die sterfft, die waken soude so dat volck zere verstorven was ende vele wt der stat waeren*”.
16. Van der Weyde, ‘Bijdrage tot de geschiedenis der pest’, p. 3120.
17. BHIC, Kloosters Mariënkroon en Mariëndonk in Heusden, 1245-1631, 239, no 87.
18. R. van de Ven, ‘Een pestordonnantie van het Diestse stadsbestuur (1483)’, *Ons Heem*, 26 (1972), pp. 148-51.
19. Van Schevensteen, ‘Over pestepidemiën’, p. 1057. New ordinance on 10 July 1484.
20. K. Dombrecht, ‘Plattelandsgemeenschappen, lokale elites en ongelijkheid in het Vlaamse kustgebied (14^de^-16^de^ eeuw)’ (unpub. Ph.D. thesis, Univ. Antwerp, 2014), pp. 81-2. There was a clear increase in the number of deceased parents in 1483 and 1484 compared to previous and later years. Actual source: RB, Registers Brugse Vrije, nos 16469-81.
21. Van Dalen, ed., ‘Oude maatregelen’, p. 127; plague ordinance (1482).
22. GA, Stadsrekening Arnhem, 1483/4, fo. 28; “*om die pestilence wil ind duer tijt*”.
23. Oosterbaan, ed., ‘Kroniek’, pp. 232-3; “*Anno LXXXIIII omtrent Paeschen begant seer te sterven van der pestilencien*” (1484).
24. J. van Veen, ed., ‘Keuren en buurspraken van Tiel en Sandwijk’, *Verslag en Mededelingen Oud Vaderlandsch Recht*, 5 (1908), pp. 359-60; “*Dat niemand in den dorpe van Santwyck eenyge vremden siecken van der pestilentiën aenemen solden in zynnen huyssen te verwaeren*” (3 May 1483).

**1487-90**

| **Region** | **Quantifiable evidence of raised mortality** | **Descriptive mentions of plague in the contemporary sources** | **Descriptive mentions of plague in later sources** |
| --- | --- | --- | --- |
| Br; Fl; Na; Ha; Ho; Me; Ar | [Flanders] (3); [Hainaut] (5); Brussels (12) | Antwerp (1); Heesbeen (2); Gouda (6); Gorinchem (7); Mechelen (8); Namur (14); Brussels (15); Ypres (16); Turnhout (13) | Namur (4); Brussels (9); Louvain (10); Ghent (11); [Brabant] (17) ; [Holland] (18) |

1. L. Torfs, *Nieuwe geschiedenis van Antwerpen*, II (Antwerp, 1865), p. 154. In 1487 it was noted that “*begonst men tot Antwerpen seer te sterven van de peste, maer doen er briefkens van den H. Naem Jesus voor byna elck huys geset wirden, dan cesseerde terstont de peste...*”.
2. BHIC, Kloosters Mariënkroon en Mariëndonk in Heusden, 1245-1631, 239, no 1463.
3. Blockmans, ‘The social and economic effects of plague’, p. 854.
4. Galliot, *Histoire Générale*, no. 1489; « *Une peste désola cette année une grande partie des Pays-Bas. Elle fut si cruelle que pendant l’espace de dix-sept mois, qu-elle dura, elle moissonna plus cinquante mille personnes dans Namur, Bruxelles, Louvain et les lieux circonvoisins* ».
5. Blockmans, ‘The social and economic effects of plague’, p. 854.
6. L. Rollin Couquerque and A. Meerkamp van Embden, eds., *Rechtsbronnen der stad Gouda* (The Hague, 1917), pp. 170-3.
7. Van Andel, ‘Pestepidemieën’.
8. F. Berlemont, ed., *Mechelse kronieken van het jaar 1 tot 1945* (Brussel, 1975).
9. Galliot, *Histoire Générale*, no. 1489; See fn. 4.
10. Ibid*.,* no. 1489; See fn. 4.
11. Van Heule, ed., *Memorieboek*, I, p. 354; “*In dit jaer storven binnen Ghendt van der peste xl duusent persoonen onder jonck ende audt*” (1489).
12. Cuvelier, ed., *Les dénombrements de foyers*, nos CCII-CCIII. Based on reduction of hearth counts, coupled with two events. First of all, the Duke of Saxony accompanied by a large group of nobles and 2000 German infantry entered Brussels on 25 August 1489, but rushed away soon after for fear of decimation by the raging disease. Second, the Brussels magistrate requested a consultation with four famous doctors to find out the best ways to combat the plague in the city. Actual source (for second example): ARB, Cartulaire et Manuscrits, no. 71, fo. 75.
13. Adriaensen, ‘Turnhout’; the sheriff’s accounts mention the plague twice in 1690; described as the “*haestige sieckte*” and “*besiect van der gaven van drie santen*”.
14. Torfs, *Fastes*, II, p. 214.
15. Ibid., II, p. 214.
16. Ibid., II, p. 214.
17. P. van Doornick, ed., *Geldersche kronieken*, II (Arnhem, 1908), p. 62; “*Was seer groote sterfte van den peste in Brabant, Holland ende ander plaetsen*” (1489).
18. Ibid., 62.

**1493-4**

| **Region** | **Quantifiable evidence of raised mortality** | **Descriptive mentions of plague in the contemporary sources** | **Descriptive mentions of plague in later sources** |
| --- | --- | --- | --- |
| Ho; Br; Ov; Gu; LvA; Ne; Fr; Ze; Fl; Ar | Leiden (1); Oudenaarde (7); Middelburg (20); Bruges (22) | Antwerp (2); Utrecht (8); Haarlem (10); Amsterdam (11); Gouda (18); Heusden (4); Lille (3); Zutphen (21); Sluis (23) | Tiel (5); Amersfoort (9); Hoorn (6); Ede; (12); Barneveld (13); Nijkerk (14); Doesburg (15); Harderwijk (16); Elburg (17); Frisia (19) |

1. Ladan, *Gezondheidszorg*, p. 59.
2. H. Dupont, ‘Sie eens ‘t verzwakte volk. De bestrijding van pest in Antwerp tijdens de vijftiende en de zestiende eeuw’, *Stadsgeschiedenis*, 2 (2007), p. 89.
3. ADN, 56 H, no. 34. Document of the *Sœurs Noires* or Augustines of Lille on the construction of “*retraits*” to evade the spread of plague, dated from 1496.
4. BHIC, Kloosters Mariënkroon en Mariëndonk in Heusden, 1245-1631, 239, no 312. (In 1495).
5. E. Dirk Rink, *Beschrijving der stad Tiel* (Tiel, 1836), p. 48.
6. T. Velius, *Chronyk van Hoorn* (Hoorn, 1740), p. 161.
7. Thoen, *Landbouwekonomie*, pp. 1141-53.
8. HUA, Stadsbestuur, 1, no. 16.
9. Van Rootselaar, *Amersfoort*, p. 35.
10. A. Enschedé (ed.), *Index op de keur- en gebodsregisters der stad Haarlem* (The Hague, 1875), pp. 55-6.
11. L. Noordegraaf and G. Valk, *De Gave Gods. De pest in Holland vanaf de late Middeleeuwen* (Bergen, 1988), p. 225.
12. Dirk Rink, *Beschrijving der stad Tiel*, p. 48.
13. Ibid., p. 48.
14. Ibid., p. 48.
15. Ibid., p. 48.
16. Ibid., p. 48.
17. Ibid., p. 48.
18. Rollin Couquerque and Meerkamp van Embden, eds., *Rechtsbronnen*, p. 170.
19. H. Nijboer, ‘De Slaande Ingel gie om de Aldehou: Eat oer de pest yn Ljouwert’, *De Vrije Fries*, 75 (1995), pp. 62-3.
20. Van den Driest, ‘Hondenbaan’, p. 38; 630 dogs killed in 1492.
21. RAZ, Inventaris van de collectie Johannes Gimberg (1850-1930), 0142, no. 68, fo. 1; priest observes special service on account of the plague.
22. SB, Hallegeboden, I, nos 104, 108, 113, 115, 121, 124, 128, 134. Seven hallegeboden (city regulations) announced on the subject of plague in 1492, and three in 1493.
23. SB, Hallegeboden, I, nos 104, 108. Noted in Bruges hallegeboden that a new outbreak of plague had occurred in August 1492, and thus a number of new restrictions on trade and mobility of Bruges citizens was now applied.

**Loose years**

**1376**

| **Region** | **Quantifiable evidence of raised mortality** | **Descriptive mentions of plague in the contemporary sources** | **Descriptive mentions of plague in later sources** |
| --- | --- | --- | --- |
| Z | [Zeeland] (1) |  |  |

1. ELO, Archief van de Sint Pancraskerk, Antiquum Registruum A, no.415, fo. 51; reference to mass mortality in Zeeland in 1376 along with sighting of a comet.

**1387-9**

| **Region** | **Quantifiable evidence of raised mortality** | **Descriptive mentions of plague in the contemporary sources** | **Descriptive mentions of plague in later sources** |
| --- | --- | --- | --- |
| Li; Na; Gu |  | [Guelders] (3) | Liege (1); Namur (2) |

1. Bacha, ed., *La chronique liégeoise*, p. 404; “*pestis super cunctum populum, tussis et raucitas*”.
2. Ibid, p. 404.
3. van Veen, ‘De pest’, p. 3; products such as pills and apples bought to counter ‘*pestilentiam*’ (1389/90).

**1393-4**

| **Region** | **Quantifiable evidence of raised mortality** | **Descriptive mentions of plague in the contemporary sources** | **Descriptive mentions of plague in later sources** |
| --- | --- | --- | --- |
| Ho | Leiden (1) |  |  |

1. Ladan, *Gezondheidszorg*, p. 45.

**1397**

| **Region** | **Quantifiable evidence of raised mortality** | **Descriptive mentions of plague in the contemporary sources** | **Descriptive mentions of plague in later sources** |
| --- | --- | --- | --- |
| Ne |  |  | Utrecht (1) |

1. *Monumenta-handschriften* of A. van Buchel, ‘Inscriptiones’, <[http://www.hetutrechtsarchief.nl/collectie/handschriften/buchelius/inscriptiones/003#_edn4](http://www.hetutrechtsarchief.nl/collectie/handschriften/buchelius/inscriptiones/003#_edn4)>, p. 3; “*Andreas ille peste sublatus est anno".*

**1407-8**

| **Region** | **Quantifiable evidence of raised mortality** | **Descriptive mentions of plague in the contemporary sources** | **Descriptive mentions of plague in later sources** |
| --- | --- | --- | --- |
| Ar | St.-Omer (1) |  |  |

1. Based on the number of *aveux* and counts served by the vassals of St. Bertin from 1403 to 1411. In a succession per year the figures were 3, 0, 1, 14, 39, 19, 11, 1 and 3; therefore the mortality was severe from 1406 to 1408. Haignère and Bled*,* eds., *Les chartes de Saint-Bertin*, nos 2168-2411.

**1431**

| **Region** | **Quantifiable evidence of raised mortality** | **Descriptive mentions of plague in the contemporary sources** | **Descriptive mentions of plague in later sources** |
| --- | --- | --- | --- |
| Fr |  | Weidum (1) |  |

1. Nijboer, ‘De Slaande Ingel’, pp. 62-3; chronicle reference from 1431.

**1476**

| **Region** | **Quantifiable evidence of raised mortality** | **Descriptive mentions of plague in the contemporary sources** | **Descriptive mentions of plague in later sources** |
| --- | --- | --- | --- |
| Fr |  | Louvain (1) |  |

1. van Even, ed., ‘Reglement’, p. 135; plague ordinance from 1473 reannounced in 1476.

**1486**

| **Region** | **Quantifiable evidence of raised mortality** | **Descriptive mentions of plague in the contemporary sources** | **Descriptive mentions of plague in later sources** |
| --- | --- | --- | --- |
| Br |  | [Taxandria] (1) |  |

1. Van Bussel, ‘De pest’, p. 168; Jacops Wautgers loses his wife and children to plague in 1486.

**1496-9^[[2]](#footnote-1)^**

| **Region** | **Quantifiable evidence of raised mortality** | **Descriptive mentions of plague in the contemporary sources** | **Descriptive mentions of plague in later sources** |
| --- | --- | --- | --- |
| Li; Ze; Fl | Middelburg (2) | Diest (1); Lille (3) |  |

1. Ordinance mentioned in Backer, ‘Maatregelen’, p. 184.
2. Van den Driest, ‘Hondenbaan’, p. 38; 1165 dogs killed in 1497.
3. ADN, 56H, 34; constrution of isolated housing to escape plague contagion from the Archive Soeurs Noires of Lille (1496).

1. We are grateful to Remi van Schaîk for access to his ‘*calamiteiten kalender*’ that pointed us to a number of references to plague in the Northern Netherlands that we had not discovered previously. [↑](#footnote-ref-0)
2. The ‘Spanish’ or ‘Naples Pox’ (syphilis) broke out in 1497, in for example, Gorinchem; W.F. Emck, ‘De voormalige pesthuizen te Gorinchem’, *Nederlands Tijdschrift voor Geneeskunde*, 73.2 (1929), p. 3571. The pox (pokken) was noted in 1496 in the hallegeboden of Bruges: SB, Hallegeboden, I, nos. 238-9. [↑](#footnote-ref-1)
